# Medicaid Value-Based Payments and Health Care Use for Patients With Mental Illness

**DOI:** 10.1001/jamahealthforum.2023.3197

**Published:** 2023-09-22

**Authors:** Ashley Lewis, Renata E. Howland, Leora I. Horwitz, Sunita M. Desai

**Affiliations:** 1 Department of Population Health, NYU Grossman School of Medicine, New York, New York; 2 Division of Healthcare Delivery Science, Department of Population Health, NYU School of Medicine, New York, New York

## Abstract

**Importance:**

Medicaid patients with mental illness comprise one of the most high-need and complex patient populations. Value-based reforms aim to improve care, but their efficacy in the Medicaid program is unclear.

**Objective:**

To investigate if New York state’s Medicaid value-based payment reform was associated with improved utilization patterns for patients with mental illness.

**Design, Setting, and Participants:**

This retrospective cohort study used a difference-in-differences analysis to compare changes in utilization between Medicaid beneficiaries whose outpatient practices participated in value-based payment reform and beneficiaries whose practices did not participate from before (July 1, 2013-June 30, 2015) to after reform (July 1, 2015-June 30, 2019). Participants were Medicaid beneficiaries in New York state aged 18 to 64 years with major depression disorder, bipolar disorder, and/or schizophrenia. Data analysis was performed from April 2021 to July 2023.

**Exposure:**

Beneficiaries were exposed to value-based payment reforms if their attributed outpatient practice participated in value-based payment reform at baseline (July 1, 2015).

**Main Outcomes and Measures:**

Primary outcomes were the number of outpatient primary care visits and the number of behavioral health visits per year. Secondary outcomes were the number of mental health emergency department visits and hospitalizations per year.

**Results:**

The analytic population comprised 306 290 individuals with depression (67.4% female; mean [SD] age, 38.6 [11.9] years), 85 105 patients with bipolar disorder (59.6% female; mean [SD] age, 38.0 [11.6] years), and 71 299 patients with schizophrenia (45.1% female; mean [SD] age, 40.3 [12.2] years). After adjustment, analyses estimated a statistically significant, positive association between value-based payments and behavioral health visits for patients with depression (0.91 visits; 95% CI, 0.51-1.30) and bipolar disorder (1.01 visits; 95% CI, 0.22-1.79). There was no statistically significant changes to primary care visits for patients with depression and bipolar disorder, but value-based payments were associated with reductions in primary care visits for patients with schizophrenia (−1.31 visits; 95% CI, −2.51 to −0.12). In every diagnostic population, value-based payment was associated with significant reductions in mental health emergency department visits (population with depression: −0.01 visits [95% CI, −0.02 to −0.002]; population with bipolar disorder: −0.02 visits [95% CI, −0.05 to −0.001]; population with schizophrenia: −0.04 visits [95% CI, −0.07 to −0.01]).

**Conclusions and Relevance:**

In this cohort study, Medicaid value-based payment reform was statistically significantly associated with an increase in behavioral health visits and a reduction in mental health emergency department visits for patients with mental illness. Medicaid value-based payment may be effective at altering health care utilization in patients with mental illness.

## Key Points

**Question:**

How are Medicaid value-based payments associated with utilization for patients with mental illness?

**Findings:**

In a difference-in-differences cohort analysis of Medicaid beneficiaries with mental illness in New York state, value-based payments were associated with a statistically significant increase in the number of yearly behavioral health visits for patients with depression (0.91 visits) and bipolar disorder (1.01 visits) and a statistically significant reduction in the number of yearly mental health emergency department visits for patients with depression (−0.01 visits), bipolar disorder (−0.02 visits), and schizophrenia (−0.04 visits).

**Meaning:**

Medicaid value-based payment may be effective at altering utilization in patients with mental illness.

## Teaser

This cohort study investigates the association of New York state’s Medicaid value-based payment reform with utilization patterns in patients with mental illness.

## Introduction

State Medicaid programs are increasingly turning to value-based payment (VBP) reform. Value-based payment programs financially incentivize clinicians and health systems to improve care. Medicaid patients experience significant health inequities, including worse access to primary care and high rates of emergency department (ED) utilization.^1,2,3,4,5,6,7^ Medicaid patients with mental health diagnoses face even worse outcomes. In a 7-year period, Medicaid patients with mental illness had a 4-times higher mortality rate than the general Medicaid population.^7,8,9^ Medicaid VBP programs aim to improve outcomes for patients with mental illness by incentivizing delivery reform that streamlines this population’s complex care.

The joint state-federal Delivery System Reform Incentive Payment (DSRIP) program is the most expansive effort to implement Medicaid VBPs to date. By 2020, 12 states had pursued or were pursuing DSRIP contracts, amounting to over $55.4 billion in joint state-federal funding.^10^ The DSRIP programs create projects to directly incentivize health systems to pursue value improvement.^11,12,13^

Despite substantial investments in Medicaid delivery and payment reform, there is limited evidence on its impacts. Early studies reported that Medicaid VBP transformation reduced primary care visits, ED visits, and hospitalizations.^14,15,16,17^ However, these studies either relied on pre-post comparisons or did not examine patients with mental illness specifically.

Using a difference-in-differences design, we analyzed the association of VBPs with utilization for Medicaid patients with mental illness in New York (NY) state. New York has the second largest Medicaid program in the nation, making its DSRIP program one of the largest implementations of Medicaid VBP reforms to date. New York’s DSRIP program also placed special emphasis on mental health care and can illustrate how VBP programs affect care for high-need populations.

### NY State DSRIP

New York state’s DSRIP proposal was approved in 2014 for $8 billion and aimed to improve patient outcomes via VBPs.^18^ The state’s DSRIP program created 25 provider networks similar to accountable care organizations. The networks comprised outpatient practices and hospitals whose collective progress on quality metrics could result in bonus payments.^19^ The networks engaged in a variety of VBP-funded population-specific or systemwide delivery reform projects, such as integration of medical and mental health care. If a clinical practice was a member of a DSRIP network, then their patient population was exposed to this delivery reform. All DSRIP networks were required to implement at least 1 behavioral health project.^12,18^ The DSRIP networks were financially rewarded if their patient population achieved milestones on specific measures, a VBP model known as pay-for-performance.^18,20^ These were bonuses in addition to the regular rates practices received via their contracts with the state (fee-for-service) or with managed care organizations.

### Study Data and Methods

We used comprehensive NY state Medicaid fee-for-service claims and encounter data and a difference-in-differences design to assess the associations of NY DSRIP program’s VBP reform with outpatient utilization and mental health ED visits and hospitalizations for Medicaid patients with mental health diagnoses.

### Data and Study Population

We used Medicaid administrative claims and encounter data, which contained enrollment characteristics, health care utilization, and diagnoses for all NY state Medicaid beneficiaries, both managed care and fee-for-service. Second, we obtained data from the NY Department of Health, which identified all national provider identifiers affiliated with DSRIP networks. The data were self-reported by DSRIP networks, who submitted formal documentation of partnership with practices.^21^ The study period was July 1, 2013, to July 1, 2019, allowing for 2 years before and 4 years after VBP implementation on July 1, 2015.

Our study population comprised non-Medicare adults (aged 18-64 years as of July 1, 2013) enrolled in Medicaid with either depression, bipolar disorder, or schizophrenia (section 3 of eMethods in Supplement 1). These diagnoses are categorized as serious mental illnesses. Patients with serious mental illnesses were a specific target population within NY DSRIP.^22^ Our diagnostic populations were not mutually exclusive. For example, if a patient was diagnosed with both major depression disorder and schizophrenia, they were included in the analyses for both diagnoses. See eMethods, section 3, for more detail on diagnostic criteria and eTable 24 in Supplement 1 for results correcting for multiple comparisons bias that could arise from the overlapping populations. Enrollees were included in the population if they had 12 months of continuous Medicaid enrollment starting on July 1 of a given study year. After exclusion, we retained 79.5% of the total population’s enrollee-months. Patients could contribute up to 6 years to the analysis if they were continuously enrolled for the entirety of the study period, with a minimum of 1 year. We also required enrollees to have at least 1 outpatient claim in the pre-VBP period for attribution purposes. Following previous studies, we attributed each enrollee to an outpatient practice based on the plurality of their outpatient visits during the 2 years prior to VBP implementation.^23,24^ We attributed patients at the practice level because if a practice was a member of a DSRIP network, its patient panel was exposed to VBP. Control practices were not DSRIP network members; therefore, their patient panel was not exposed to VBP. This procedure is further described in section 2 of eMethods in Supplement 1. The NYU Langone Health Institutional Review Board approved this as a human research study, with approval for waiver of consent. This study followed the Strengthening the Reporting of Observational Studies in Epidemiology (STROBE) reporting guideline for cohort studies.

### VBP Participation

A practice was defined as a VBP participant if it was affiliated with a DSRIP network in the first year of VBP implementation, given that the majority of practices began participation in the first year and the attrition rate out of VBP is very low (<1%). We refer to these as VBP practices. However, in sensitivity analyses, we allow for time-varying VBP participation (sections 4 and 8 of eMethods and eTables 4-6 and 7 in Supplement 1). We compare baseline characteristics of VBP and non-VBP practices in eTable 1 in Supplement 1.

### Outcomes

Our primary outcomes were the number of behavioral health outpatient visits and the number of primary care visits in a year. See section 6 of eMethods in Supplement 1 for more details. These outcomes align with the NY DSRIP program’s explicit goal to increase outpatient care as a means to reduce high-cost utilization.^25^ Many NY DSRIP delivery reform projects focused on transforming the outpatient setting.^16^ Outpatient utilization is considered an important outcome in evaluations of other mental health programs.^26,27,28^ In eTable 22 in Supplement 1, we examined the net effects on outpatient visits. Additionally, since primary care is an important delivery site for mental health services,^29^ we examined in a secondary analysis how VBP was associated with the number of mental health–related primary care visits (eTable 23 in Supplement 1).

As secondary outcomes, we examined the number of mental health hospitalizations and ED visits in a year. Mental health hospitalizations and ED visits were identified by admission with a primary diagnosis related to mental health (section 3 of eMethods in Supplement 1). We examined these outcomes because reducing mental health hospitalizations and ED use was a top priority for NY DSRIP.^25^ These measures are also correlated with relevant measures of health and are common proxies for health outcomes not available in administrative data.^30,31^ Details on construction of the outcomes are in sections 3 and 6 of eMethods in Supplement 1. In secondary analyses, we examined changes in all-cause hospitalizations and ED visits to measure VBP’s association with overall high-cost utilization (eTable 3 in Supplement 1). We also examined readmissions and preventable hospitalizations to measure VBP’s association with quality (eTable 21 in Supplement 1).

### Statistical Analysis

We used a difference-in-differences research design, comparing outcomes of patients attributed to VBP participating practices to outcomes of patients attributed to non-VBP participating practices before vs after the implementation of NY’s Medicaid VBP reform in July 2015. Our model included year fixed effects, patient fixed effects, and the interaction of indicators representing the post-VBP period and whether a patient was attributed to a VBP practice. Patient and year fixed effects were collinear with individual indicators (not included in the interaction term) for the post-VBP period and attribution to a VBP practice, so the indicators were excluded from our model. Patient fixed effects account for whether the patient was attributed to a VBP practice as well as time-invariant observable and unobservable patient-level factors. Although patient fixed effects account for time-invariant individual differences, we compared baseline patient characteristics in Table 1 to describe the VBP and non-VBP populations. Year fixed effects account for observable and unobservable characteristics that change each year across all patients, including post-VBP status. The explanatory variable of interest was the interaction between indicators for the post-VBP period and attribution to a VBP practice. The estimated coefficient for this variable represented the difference-in-differences estimate or the adjusted difference in outcomes from before to after VBP implementation for patients attributed to VBP practices compared with the difference in outcomes for patients attributed to non-VBP practices during the same time period. We estimated linear models in the main analysis and tested alternate functional forms in secondary analyses. We estimated heteroskedasticity-robust standard errors clustered at the practice level.

**Table 1.** Patient Characteristics in the Pre-VBP Period (July 1, 2013-June 30, 2015) Stratified by Diagnosis and VBP Attribution

Characteristic	Depression			Bipolar disorder			Schizophrenia		
	Total	VBP	Non-VBP	Total	VBP	Non-VBP	Total	VBP	Non-VBP
Total No.	306 290	244 705	61 585	85 105	70 272	14 832	71 299	58 751	12 548
Age, mean (SD), y	38.6 (11.9)	38.7 (11.9)	38.4 (11.7)	38.0 (11.6)	38.1 (11.7)	37.8 (11.6)	40.3 (12.2)	40.3 (12.2)	40.4 (12.2)
Sex, %									
Female	67.4	67.3	65.9	59.6	59.6	59.2	45.1	45.3	44.3
Male	32.6	32.7	34.1	40.4	40.4	40.8	54.9	54.7	55.7
Race and ethnicity, %^a^									
Asian	3.9	3.4	6.2	2.4	2.2	3.7	4.5	4.2	6.4
Black	17.6	18.7	12.5	21.8	22.5	17.2	30.7	31.4	26.8
Native American	4.1	4.2	3.8	4.5	4.5	4.1	5.5	5.6	5.1
Hispanic	17.4	18.8	11.2	19.7	20.9	13.4	20.7	21.6	15.2
White	41.2	39.0	51.03	39.5	37.7	49.2	27.8	26.6	35.4
Total enrollment, mean (SD), mo	53.1 (20.7)	53.6 (20.6)	57.8 (21.4)	56.5 (19.6)	56.8 (19.5)	55.1 (20.2)	58.3 (19.3)	58.5 (19.2)	57.0 (20.0)
Managed care, %	96.7	96.4	97.8	94.5	94.2	96.0	90.3	90.0	92.3
Charlson index, mean (SD)^b^	2.1 (3.1)	2.2 (3.1)	1.7 (2.8)	2.7 (3.4)	2.7 (3.4)	2.6 (3.3)	2.9 (3.6)	2.9 (3.6)	2.9 (3.6)

Abbreviation: VBP, value-based payment.

^a^ All racial and ethnic categories are non-Hispanic except the Hispanic category. Percentages for racial identity do not add up to 100% due to missing values. The missingness of race, stratified by diagnosis and VBP status, are the following: depression, VBP: 15.9%; non-VBP: 15.3%; bipolar disorder, VBP: 12.2%; non-VBP: 12.0%; schizophrenia, VBP: 10.7%; non-VBP: 11.3%.

^b^ Average comorbidity score is the average patient-level Charlson comorbidity index score in the pre-VBP period. Charlson comorbidity index is a comorbidity measure to predict mortality within 1 year and is used as a proxy for disease burden. A diagnosis was included for calculation if it was one of 16 chronic illnesses listed for the Charlson comorbidity index and was reported in any patient diagnostic variable between July 1, 2013, and June 30, 2015. Higher scores represent more severe burden of disease, with a range of 0 to 24.

A key assumption underlying our model was that, in the absence of VBPs, outcomes for patients served by VBP practices would have trended similarly to the outcomes of patients served by non-VBP practices. To examine the validity of this assumption, we statistically tested and visually examined whether outcomes for the VBP and non-VBP populations trended similarly prior to VBP at the year and 6-month level. We also tested the robustness of our main results by adjusting for any pre-VBP trend differences between VBP and non-VBP patients (eTables 8-11 in Supplement 1). We tested for compositional differences in patient characteristics throughout our study period that could bias our estimates (eTable 12 in Supplement 1). In a sensitivity analysis, we also limited the analysis to enrollees who were enrolled throughout the entire study period, eliminating potential bias due to compositional differences between VBP and non-VBP groups (eTables 17 and 18 in Supplement 1).

We conducted several analyses (section 8 of eMethods in Supplement 1) to test the robustness of our results. First, we tested the robustness of our main results to additional functional forms (eTables 13 and 14 in Supplement 1). Second, to test the degree to which differential selection based on enrollment could produce bias, we limited the analysis to a continuously enrolled sample of patients. Third, we attributed patients based on their pre-VBP care, but a patient’s primary practice could change from year to year. To mitigate bias and noise from such changes, we restricted our population to patients with the same VBP exposure throughout the study period (eTables 19 and 20 in Supplement 1). Fourth, we investigated if VBP was associated with utilization changes from no use to any use (extensive margin) and/or in the intensity of utilization (intensive margin) using a 2-part model (eTables 15 and 16 in Supplement 1). Fifth, we stratified analyses by enrollees residing in New York City (NYC) vs non-NYC (eTables 25 and 26 in Supplement 1), and, for patients with schizophrenia, we stratified by comorbidity score (eTable 27 in Supplement 1).^32^

See the eMethods in Supplement 1 for more details. Analyses were conducted using Stata, version 15 (StataCorp LLC). Statistical significance was set at *P* < .05, and all tests were 2-sided.

## Results

### Population Characteristics

To describe VBP and non-VBP patient populations, we report average patient characteristics for each group, stratified by diagnosis, in Table 1. The analytic population comprised 306 290 patients with depression (79.9% VBP; 67.4% female; mean [SD] age, 38.6 [11.9] years), 85 105 patients with bipolar disorder (82.6% VBP; 59.6% female; mean [SD] age, 38.0 [11.6] years), and 71 299 patients with schizophrenia (82.4% VBP; 45.1% female; mean [SD] age, 40.3 [12.2] years). The percentage of patients who were non-Hispanic White was somewhat different between VBP and non-VBP patients (depression: 39.0% VBP vs 51.0% non-VBP; bipolar disorder: 37.7% VBP vs 49.2% non-VBP; schizophrenia: 26.6% VBP vs 35.4% non-VBP). Average total enrollment, average Charlson comorbidity index scores, percentage by sex, average age, and managed care participation were similar between VBP and non-VBP groups. Patients analyzed were similar to patients excluded due to insufficient enrollment (eTable 2 in Supplement 1).

### Behavioral Health and Primary Care Outpatient Visits

For patients with depression, VBP was associated with a statistically significant increase of 0.91 (95% CI, 0.51-1.30) behavioral health visits, after adjustment. After adjustment, VBP was associated with a relative decrease of primary care visits for VBP patients, though this was a nonsignificant change. For the population with bipolar disorder, VBP was associated with a statistically significant increase of 1.01 (95% CI, 0.22-1.79) behavioral health visits, after adjustment, and a nonsignificant relative decrease in primary care visits. For patients with schizophrenia, VBP was associated with a nonsignificant increase in behavioral health visits but a statistically significant decrease in primary care visits (−1.31 visits; 95% CI, −2.51 to −0.12) (Table 2). When the population with schizophrenia was stratified by comorbidity score, we found that VBP-associated reductions in primary care were driven by patients with the fewest comorbidities (eTable 27 in Supplement 1). In a secondary analysis, we found that VBP-attributed patients with depression and bipolar disorder had relative increases in mental health–related primary care visits, while patients with schizophrenia had no significant change (eTable 23 in Supplement 1).

**Table 2.** Primary Outcomes: Differential Change in Outcomes After VBP for VBP-Attributed Patients

Outcome	Mean visits per year^a^				Change in No. visits per year			
	VBP		Non-VBP		Unadjusted^b^		Adjusted^c^	
	Before VBP	After VBP	Before VBP	After VBP	Estimate (95% CI)	*P* value	Estimate (95% CI)	*P* value
**Depression**								
Behavioral health	3.6	5.8	3.9	5.3	0.87 (0.49 to 1.25)	<.001	0.91 (0.51 to 1.30)	<.001
Primary care	8.5	8.8	6.5	7.0	−0.23 (−0.76 to 0.30)	.40	−0.36 (−0.89 to 0.16)	.18
**Bipolar disorder**								
Behavioral health	6.1	9.0	6.7	8.6	0.96 (0.21 to 1.71)	.01	1.01 (0.22 to 1.79)	.01
Primary care	9.7	11.2	6.9	8.9	−0.5 (−1.45 to 0.45)	.30	−0.71 (−1.70 to 0.28)	.16
**Schizophrenia**								
Behavioral health	8.0	9.4	7.8	9.1	0.17 (−0.73 to 1.07)	.72	0.26 (−0.72 to 1.23)	.61
Primary care	9.5	10.8	6.5	9.0	−1.13 (−2.27 to 0.01)	.05	−1.31 (−2.51 to −0.12)	.03

Abbreviations: NPI, national provider identifier; VBP, value-based payment.

^a^ Visits were identified as an aggregation of claims per patient-NPI-day so that all claims associated with 1 visit were counted as a single visit. To categorize visits, we used a Category of Service variable derived from an algorithm of NPI specialty codes, place of service variables, procedure codes, and rate codes.

^b^ Unadjusted models included an indicator for patient attribution to a VBP practice, an indicator for being in the post-VBP period, and the interaction between these 2 indicators. Standard errors were clustered at the practice level.

^c^ Adjusted models included the interaction between indicators for the post-VBP period and being attributed to a post-VBP practice and individual patient and year fixed effects. Standard errors were clustered at the practice level.

### Mental Health Hospitalizations and ED Visits

For the population with depression, after adjustment, VBP was associated with a small, statistically significant decrease in mental health hospitalizations (−0.01 visits; 95% CI, −0.01 to −0.004). In every diagnostic population, VBP was associated with a significant reduction in mental health ED visits (depression: −0.01 visits [95% CI, −0.02 to −0.002]; bipolar disorder: −0.02 visits [95% CI, −0.05 to −0.001]; schizophrenia: −0.04 visits [95% CI, −0.07 to −0.01]) (Table 3). In secondary analyses, we found that all-cause hospitalizations and ED visits were directionally consistent with mental health admission results (eTable 3 in Supplement 1). As shown in eTable 21 in Supplement 1, we found that VBP was associated with reductions in readmissions and preventable hospitalizations, but not significantly.

**Table 3.** Secondary Outcomes: Differential Change in Outcomes After VBP for VBP-Attributed Patients

Outcome	Mean visits per year^a^				Change in No. visits per year			
	VBP		Non-VBP		Unadjusted^b^		Adjusted^c^	
	Before VBP	After VBP	Before VBP	After VBP	Estimate (95% CI)	*P* value	Estimate (95% CI)	*P* value
**Depression**								
Mental health hospitalizations	0.08	0.06	0.06	0.06	−0.01 (−0.01 to −0.003)	<.001	−0.01 (−0.01 to −0.004)	<.001
Mental health ED visits	0.08	0.09	0.06	0.08	−0.01 (−0.01 to −0.001)	.03	−0.01 (−0.02 to −0.002)	.01
**Bipolar disorder**								
Mental health hospitalizations	0.23	0.17	0.22	0.17	0 (−0.02 to 0.01)	.77	−0.01 (−0.02 to 0.01)	.45
Mental health ED visits	0.19	0.22	0.17	0.22	−0.02 (−0.04 to 0.003)	.09	−0.02 (−0.05 to −0.001)	.04
**Schizophrenia**								
Mental health hospitalizations	0.33	0.25	0.34	0.26	0 (−0.02 to 0.02)	.72	−0.01 (−0.03 to 0.01)	.47
Mental health ED visits	0.26	0.29	0.23	0.29	−0.03 (−0.06 to 0.002)	.06	−0.04 (−0.07 to −0.01)	.02

Abbreviations: ED, emergency department; NPI, national provider identifier; VBP, value-based payment.

^a^ Visits were identified as an aggregation of claims per patient-NPI-day so that all claims associated with 1 visit were counted as a single visit. To categorize visits, we used a Category of Service variable derived from an algorithm of NPI specialty codes, place of service variables, procedure codes, and rate codes.

^b^ Unadjusted models included an indicator for patient attribution to a VBP practice, an indicator for being in the post-VBP period, and the interaction between these 2 indicators. Standard errors were clustered at the practice level.

^c^ Adjusted models included the interaction between indicators for the post-VBP period and being attributed to a post-VBP practice and individual patient and year fixed effects. Standard errors were clustered at the practice level.

### Temporal VBP Association With Outcomes

**Figure 1.**
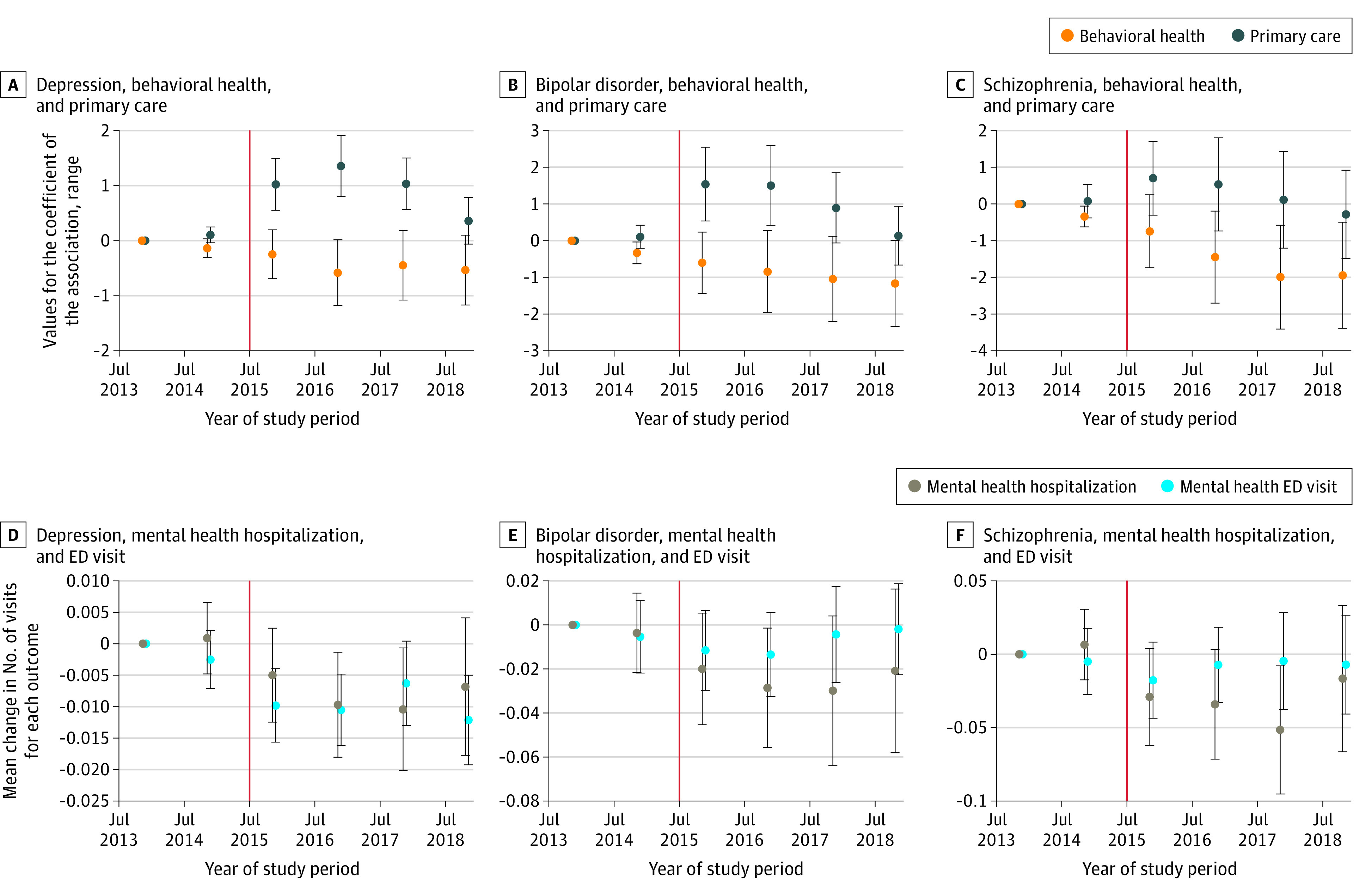
Differential Change in Outcomes for VBP Patients in Each Year The y-axis displays the range in values for the coefficients of the association between value-based payment (VBP) (A-C) and the mean change in the number of visits for each outcome (D-F). The colored dots are the estimates from the adjusted model for every year of the study, with July 1, 2013, to June 30, 2014, set as the reference year. The estimates are centered between years to indicate the midpoint between the start and end of each study year (July 1). The vertical red line represents the implementation of VBP on July 1, 2015. ED indicates emergency department.

Figure 1 displays the adjusted model estimates decomposed by year. Effect sizes of the association of VBP with behavioral health visits were largest in the first 3 postperiod years for patients with depression and bipolar disorder. For patients with depression, reductions in mental health ED visits and hospitalizations remained consistent after VBP, while patients with bipolar disorder and schizophrenia had reduced effect sizes over time.

### Robustness Test

Our robustness analyses were consistent with our main results (details in section 8 of eMethods, eFigure, and eTables 3-24 in Supplement 1). Overall, our results were robust to pre-VBP outcome trend inclusion, 2-part models, restriction to a balanced panel, log-transformed outcomes, multiple comparisons, and analyses on VBP exposure consistency. Our 2-part model showed that VBP was associated with both an increase in the likelihood of any behavioral health visits as well as an increase in the number of visits, given any utilization. When the population was set to a balanced panel, results were similar to the main findings, indicating that population compositional changes did not drive the associations. When the patient population had consistent VBP exposure, VBP was associated with an increase, rather than a decrease, in primary care visits.

### Pre-VBP Outcome Trend

**Figure 2.**
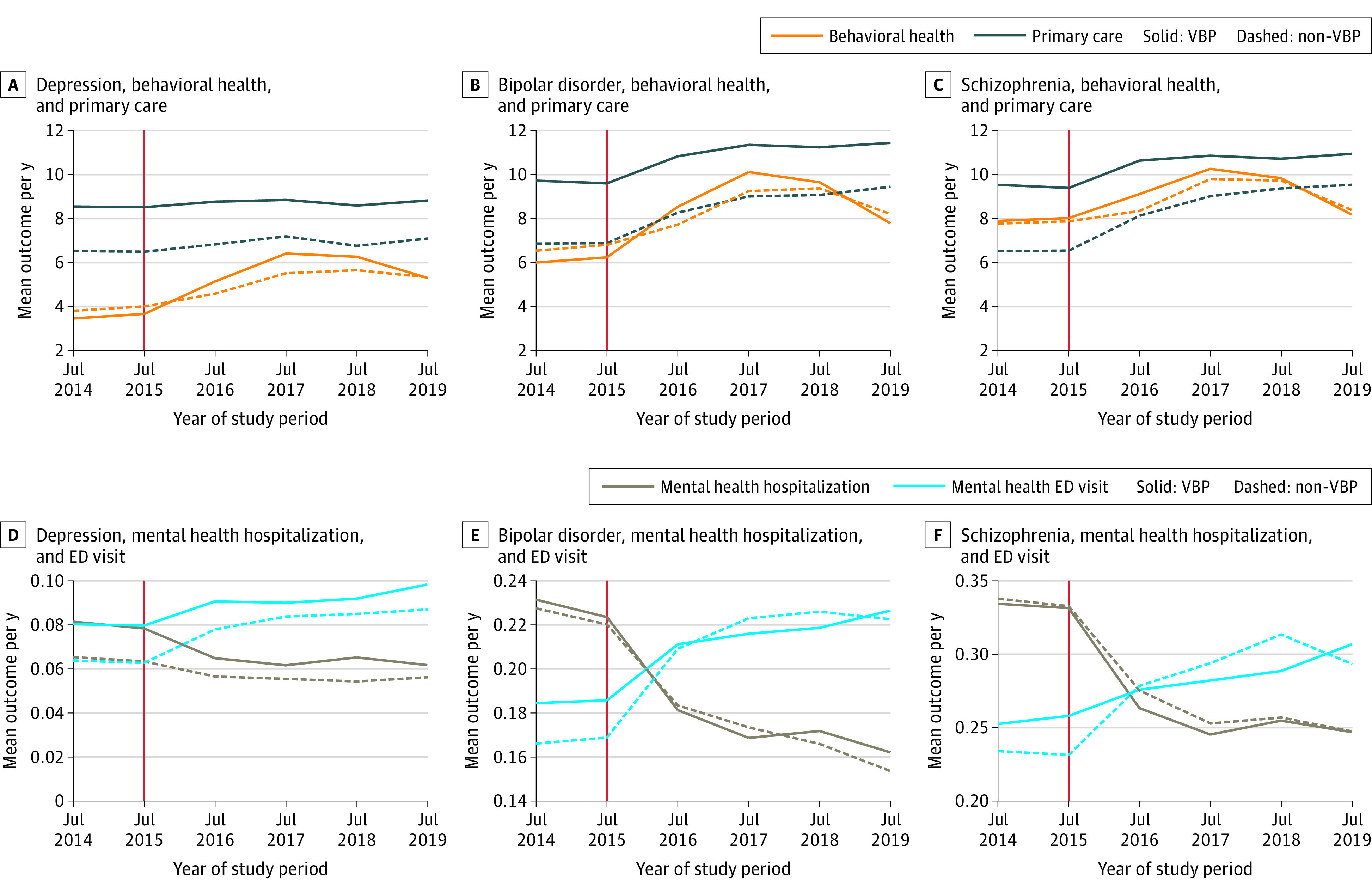
Mean Number of Visits per Year for VBP and Non-VBP Groups, Stratified by Diagnosis The y-axis is the average mean outcome per year for value-based payment (VBP) and non-VBP patient populations stratified by diagnosis. The x-axis represents each year of the study period. The 2 pre-VBP–period year averages are represented by the data points above “Jul 2014” (the average mean outcome for July 1, 2013-June 30, 2014) and the data points above “Jul 15” (the average mean outcome for July 1, 2014-June 30, 2015). The vertical red line indicates the implementation of VBP on July 1, 2015. Error bars represent 95% CIs. ED indicates emergency department.

Overall, pre-VBP outcome trends between the VBP and non-VBP groups appeared to be similar (Figure 2). In formal statistical tests, trends for all but 1 outcome were not different in the VBP and non-VBP groups (Figure 1). Primary care visits demonstrate a small differential reduction for the VBP group. After adjusting for pre-VBP trend differences, our estimates were still similar, though somewhat attenuated (eTables 8 and 9 in Supplement 1). We also examined outcome trends at the 6-month level for increased granularity (eFigure in Supplement 1). Overall, outcome trends appeared to be parallel, with the exception of mental health hospitalizations for patients with bipolar disorder and schizophrenia. Our results remained consistent when pre-trends were included compared with the general difference-in-differences model at the 6-month level (eTables 10 and 11 in Supplement 1), with mental health hospitalization showing no significant changes for patients with bipolar disorder and schizophrenia.

## Discussion

We used a difference-in-differences design to assess whether outpatient utilization and mental health hospitalizations and ED visits for Medicaid patients with mental illness changed after NY state implemented VBP reform via DSRIP. Consistent with policy goals, VBP reform was associated with more behavioral health visits and fewer mental health ED visits. Generally, the strongest and most sustained associations were seen in patients with depression.

A potential mechanism for the positive change in patient utilization we observed after NY DSRIP could be the program’s combination of VBPs with structured delivery system reform. In early years of the program, DSRIP networks were provided with funding to implement required infrastructure changes for their selected projects (eg, integrated electronic health record systems). A NY state report of DSRIP found that, among organizations participating, a majority saw positive impacts on patient care related to delivery projects.^33^ Previous evaluations of VBP programs have struggled to identify underlying delivery setting changes used by participating organizations because many VBP programs focused on payment transformation without specific delivery reform requirements.^34^ As more state Medicaid programs transition to VBPs, programs should prioritize the combination of VBP and delivery setting reform that specifically caters to the needs of patients with mental illness.

For patients with schizophrenia, we observed significant reductions in primary care visits after VBP. Since primary care can be a setting in which mental health care is delivered,^29^ this could raise concerns that VBP was associated with overall declines in mental health care delivery. However, in secondary analyses, we found that VBP did not significantly change mental health–related primary care visits (eTable 23 in Supplement 1), mitigating concerns that the reduction in overall primary care offset increases in behavioral health visits.

An empirical and substantive challenge to Medicaid VBP programs is patient churn. A concern for Medicaid VBP reform is that benefits from delivery system reforms may not be realized until the long term, which would be undermined by churn. In eTables 19 and 20 in Supplement 1, we restricted the population to patients with consistent VBP exposure throughout the study period to identify VBP’s effects within a population that has long-term VBP exposure. Notably, we found that VBP patients with consistent exposure had a statistically significant, positive association with primary care visits rather than reductions. This suggests that the strength and direction of VBP’s association with primary care depends on VBP exposure length. Nonetheless, our findings show that despite patient churn, Medicaid VBPs can change utilization patterns positively for patients with mental illness.

### Limitations

This study has several limitations. First, this analysis pertains to NY state, and findings may not generalize to other states. Still, our findings shed light on one of the largest DSRIP programs to date and could offer examples of successful program designs. Second, NY DSRIP occurred simultaneously with other reforms, including Medicaid managed care VBP contracting, that we were unable to directly observe in our data. To the extent that practices participating in project delivery–related VBP are not differentially likely to engage in managed care VBP, our results would not be biased. Third, it is possible that clinicians in our data could be practicing in both VBP and non-VBP practices, which could lead to spillover effects in our control population. However, bias is likely minimal because the majority of VBP-related delivery reform occurred at the system or clinic level that could not be transferred by individual clinicians. Fourth, secular trends in utilization may differ on a more granular geographical level than we can measure. However, we did conduct a geographical comparison of NYC vs non-NYC regions to assess differences in the association of VBP with different health care environments.

## Conclusions

In this cohort study, we found that Medicaid VBPs were associated with increased behavioral health visits and reductions in mental health ED visits for patients with mental illness. Our work contributes to a growing literature demonstrating benefits of alternative payment models within Medicaid and mental health care.

## Notes

Supplement 1.
eMethods.
**eFigure.** The Mean Number of Visits of six-month period for VBP and non-VBP **eTable 1.** Characteristics of NPIs in analytic sample by VBP participation and NPI type **eTable 2.** Pre-VBP Characteristics of Analytic Population vs. Patients Excluded for Insufficient Enrollment **eTable 3.** Differential changes in all-cause hospitalizations and ED visits for VBP patients after VBP **eTable 4.** Time-varying VBP indicator throughout study period: Differential change in primary outcomes after VBP for VBP patients **eTable 5.** Time-varying VBP indicator throughout study period: Differential change in secondary outcomes after VBP for VBP attributed patients **eTable 6.** Restricted to patients whose practice enters VBP in the First VBP year: Differential change in primary outcomes after VBP for baseline VBP patients **eTable 7.** Restricted to patients whose practice enters VBP in the First VBP year: Differential change in secondary outcomes after VBP for baseline VBP patients **eTable 8.** Incorporation of Pre-VBP outcome trends: Differential change in primary outcomes after VBP for VBP patients **eTable 9.** Incorporation of Linear Pre-VBP outcome trends: Differential change in secondary outcomes after VBP for VBP patients groups, stratified by diagnosis **eTable 10.** Differential change in outcomes for VBP patients after VBP at the six-month period level **eTable 11.** Incorporation of Pre-VBP outcome trends at the six-month period level: Differential change in primary outcomes after VBP for VBP patients **eTable 12.** Differential changes in patient characteristics for VBP vs non-VBP patients in each study year relative to July 2013-2014 **eTable 13.** Log-transformed primary outcomes: Differential change in outcomes after VBP for VBP attributed patients **eTable 14.** Log-transformed secondary outcomes: Differential change in outcomes after VBP for VBP attributed patients **eTable 15.** Two Part Model: Differential change in primary outcomes after VBP for VBP patients **eTable 16.** Two Part Model: Differential change in secondary outcomes after VBP for VBP patients **eTable 17.** Balanced Panel primary outcomes: Differential change after VBP for VBP attributed patients **eTable 18.** Balanced Panel secondary outcomes: Differential change after VBP for VBP attributed patients **eTable 19.** Restricted patient population whose VBP exposure status is consistent throughout study period: Differential change in primary outcomes after VBP for VBP attributed patients **eTable 20.** Restricted patient population whose VBP exposure status is consistent throughout study period: Differential change in secondary outcomes after VBP for VBP attributed patients **eTable 21.** Differential change in quality outcomes after VBP for VBP attributed patients **eTable 22.** Differential change in Combined Outpatient Utilization after VBP for VBP attributed patients **eTable 23.** Differential change in Mental Health Primary Care (MHPC) visits after VBP for VBP attributed patients **eTable 24.** Multiple Comparison Adjustment for primary and secondary outcomes **eTable 25.** NYC vs non-NYC: Differential change in primary outcomes after VBP for VBP patients **eTable 26.** NYC vs non-NYC: Differential change in secondary outcomes after VBP for VBP patients **eTable 27.** Differential change in outcomes after VBP for Schizophrenia VBP patients stratified by Charlson Co-Morbidity Score
eReferences

Supplement 2.
Data Sharing Statement
