# Supplementary material for: Medicaid Value-Based Payments and Health Care Use for Patients With Mental Illness
Source: JAMA Health Forum. 2023 Sep 22;4(9):e233197. doi: 10.1001/jamahealthforum.2023.3197 (PMC10517380; doi:10.1001/jamahealthforum.2023.3197)
Supplement: Supplement 1. — eMethods. eFigure. The Mean Number of Visits of six-month period for VBP and non-VBP eTable 1. Characteristics of NPIs in analytic sample by VBP participation and NPI type eTable 2. Pre-VBP Characteristics of Analytic Population vs. Patients Excluded for Insufficient Enrollment eTable 3. Differential changes in all-cause hospitalizations and ED visits for VBP patients after VBP eTable 4. Time-varying VBP indicator throughout study period: Differential change in primary outcomes after VBP for VBP patients eTable 5. Time-varying VBP indicator throughout study period: Differential change in secondary outcomes after VBP for VBP attributed patients eTable 6. Restricted to patients whose practice enters VBP in the First VBP year: Differential change in primary outcomes after VBP for baseline VBP patients eTable 7. Restricted to patients whose practice enters VBP in the First VBP year: Differential change in secondary outcomes after VBP for baseline VBP patients eTable 8. Incorporation of Pre-VBP outcome trends: Differential change in primary outcomes after VBP for VBP patients eTable 9. Incorporation of Linear Pre-VBP outcome trends: Differential change in secondary outcomes after VBP for VBP patients groups, stratified by diagnosis eTable 10. Differential change in outcomes for VBP patients after VBP at the six-month period level eTable 11. Incorporation of Pre-VBP outcome trends at the six-month period level: Differential change in primary outcomes after VBP for VBP patients eTable 12. Differential changes in patient characteristics for VBP vs non-VBP patients in each study year relative to July 2013-2014 eTable 13. Log-transformed primary outcomes: Differential change in outcomes after VBP for VBP attributed patients eTable 14. Log-transformed secondary outcomes: Differential change in outcomes after VBP for VBP attributed patients eTable 15. Two Part Model: Differential change in primary outcomes after VBP for VBP patients eTable 16. Two Part Model: Differential chang [file jamahealthforum-e233197-s001.pdf]

Supplemental Online Content

Lewis A, Howland RE, Horwitz LI, Desai SM. Medicaid value-based payments and health care use for patients with mental illness. *JAMA Health Forum.* 2023;4(9):e233197. doi:10.1001/jamahealthforum.2023.3197

eMethods.

eFigure. The Mean Number of Visits of six-month period for VBP and non-VBP

eTable 1. Characteristics of NPIs in analytic sample by VBP participation and NPI type

eTable 2. Pre-VBP Characteristics of Analytic Population vs. Patients Excluded for Insufficient Enrollment

eTable 3. Differential changes in all-cause hospitalizations and ED visits for VBP patients after VBP

eTable 4. Time-varying VBP indicator throughout study period: Differential change in primary outcomes after VBP for VBP patients

eTable 5. Time-varying VBP indicator throughout study period: Differential change in secondary outcomes after VBP for VBP attributed patients

eTable 6. Restricted to patients whose practice enters VBP in the First VBP year: Differential change in primary outcomes after VBP for baseline VBP patients

eTable 7. Restricted to patients whose practice enters VBP in the First VBP year: Differential change in secondary outcomes after VBP for baseline VBP patients

eTable 8. Incorporation of Pre-VBP outcome trends: Differential change in primary outcomes after VBP for VBP patients

eTable 9. Incorporation of Linear Pre-VBP outcome trends: Differential change in secondary outcomes after VBP for VBP patients groups, stratified by diagnosis

eTable 10. Differential change in outcomes for VBP patients after VBP at the six-month period level

eTable 11. Incorporation of Pre-VBP outcome trends at the six-month period level: Differential change in primary outcomes after VBP for VBP patients

eTable 12. Differential changes in patient characteristics for VBP vs non-VBP patients in each study year relative to July 2013-2014

eTable 13. Log-transformed primary outcomes: Differential change in outcomes after VBP for VBP attributed patients

© 2023 Lewis A et al. *JAMA Health Forum.*

eTable 14. Log-transformed secondary outcomes: Differential change in outcomes after VBP for VBP attributed patients

eTable 15. Two Part Model: Differential change in primary outcomes after VBP for VBP patients

eTable 16. Two Part Model: Differential change in secondary outcomes after VBP for VBP patients

eTable 17. Balanced Panel primary outcomes: Differential change after VBP for VBP attributed patients

eTable 18. Balanced Panel secondary outcomes: Differential change after VBP for VBP attributed patients

eTable 19. Restricted patient population whose VBP exposure status is consistent throughout study period: Differential change in primary outcomes after VBP for VBP attributed patients

eTable 20. Restricted patient population whose VBP exposure status is consistent throughout study period: Differential change in secondary outcomes after VBP for VBP attributed patients

eTable 21. Differential change in quality outcomes after VBP for VBP attributed patients

eTable 22. Differential change in Combined Outpatient Utilization after VBP for VBP attributed patients

eTable 23. Differential change in Mental Health Primary Care (MHPC) visits after VBP for VBP attributed patients

eTable 24. Multiple Comparison Adjustment for primary and secondary outcomes

eTable 25. NYC vs non-NYC: Differential change in primary outcomes after VBP for VBP patients

eTable 26. NYC vs non-NYC: Differential change in secondary outcomes after VBP for VBP patients

eTable 27. Differential change in outcomes after VBP for Schizophrenia VBP patients stratified by Charlson Co-Morbidity Score

eReferences

This supplemental material has been provided by the authors to give readers additional information about their work.

eMethods

1. Defining and characterizing practices

We define outpatient practices as billing NPIs whose values for our "Category of Service" variable are either primary care or specialty care. The Category of Service variable is produced from an algorithm which weighs an NPI specialty code, place of service codes, rate codes, and procedure codes. This algorithm is a based on the New York State Medicaid's MMCOR algorithm, which classifies types of utilization into accurate service types.[1] We use the billing NPI in the Medicaid claims data, which represents an organizational NPI for a group or solo practice or an individual NPI for a an individual physician. We identified organization type based on data from New York state. Organizations were identified in the Institutional Provider Network Data System and individual clinicians were identified in the Individual Provider Network Data System data.[2] We used data from New York Department of Health to identify safety-net clinicians and organizations.[3] Supplement eTable 1 depicts these characteristics. There are a similar proportion of individual and organizational NPIs between the VBP and non-VBP groups. VBP and non-VBP individual NPIs have similar panel sizes, while VBP organizations have larger patient panels than non-VBP organizations. VBP groups had a slightly higher proportion of Individual NPIs that were classified as primary care physicians and as safety-net. This is aligned with DSRIP's goal of recruiting safety-net and primary care clinicians. The different percentage of safety-net clinicians between the VBP and non-VBP NPI sample may explain the baseline difference we observe in the race and ethnicity composition between VBP and non-VBP groups, as safety-net providers are disproportionately more likely to care for racial minorities.[4] The patient-level fixed effects in our model in combination with the fixed patient attribution to a single NPI from the pre-VBP period account for differences in provider characteristics in our estimates.

Of the NPIs that were categorized as individuals, we randomly selected 100 NPIs and manually researched whether those NPIs belonged to individuals billing within a group practice or those of a solo practitioner. The majority (87%) were individual clinicians practicing within a group practice. Therefore, we conceptualize the billing NPI variable as a group practice.

2. Patient attribution

We used patients' outpatient utilization within the pre-VBP of July 1, 2013-July 1, 2015 to assign patients to NPIs and, ultimately, to VBP exposed or control groups. All patients with at least one month enrollment during the pre-VBP were included in the attribution. The attribution included claims from every month of enrollment in this period for each patient. Outpatient utilization was defined as any visit to an outpatient practice (see Supplemental eSection 1). If the plurality of a patient's visits were with a practice during the pre-VBP period the patient was attributed to the practice. If there was a tie, a practice was randomly chosen for assignment. If the patient's attributed practice was a participant in VBP through DSRIP, the patient will be in the VBP-exposed (treated) group. This is similar to NY DSRIP attribution logic, which assigned patients to DSRIP networks (i.e. being "treated") using the site of their most frequently used utilization. The average total outpatient claims in the pre-VBP eligible for the attribution processes is 27.8 claims for patients who are ultimately attributed to VBP and 21.4 claims for those ultimately attributed to non-VBP.

We choose to use outpatient utilization as opposed to exclusively primary care because of the particular emphasis of New York DSRIP on wide variety of clinicians. Many previous VBP programs were set exclusively within the primary care setting, therefore primary care utilization was the only utilization contributing to the attribution methodology. In contrast, NY DSRIP engaged clinicians of all specializations for both NY state DSRIP network-patient attribution methodology and for VBP project delivery reform.[5] Further, in our analytic population, 75% of the NPIs to which our patients had a plurality of outpatient visits (their attributed practice) are specialists. This highlights that our Medicaid mental health populations have a heavy-specialty driven outpatient utilization and incorporating specialists into attribution is a more accurate reflection of the setting patients were exposed to VBP.

3. **Mental Illness Diagnoses Definitions:**
   a. Defining the mentally ill population: We use the Clinical Classification Software (CCS) and ICD-9 codes to define the depression, bipolar, and schizophrenia populations. We used the following CCS codes for initial population categorization: Depression (657), Bipolar disorder (657), and Schizophrenia (659). Since CCS code 657 represents Mood disorders, which encompasses both depression and bipolar disorders, we further distinguish between these diagnostic populations using ICD-9 codes.[6]
      i. **Depression**: 3091; 30928; 29621; 311; 29622; 29635; 29626; 29632; 29623; 29630; 3004; 29634; 29636; 29682; 29624; 29625; 29633.
      ii. **Bipolar**: 29652, 29603; 29655, 29654; 29656;29616; 29662; 29665; 29640; 29689; 29613; 29666; 29614; 29614; 29615; 29660; 29606; 29604; 29600; 29601; 29646; 29653; 29650; 2967; 29644; 29644; 29651; 29643; 29681; 29664; 29663; 29611; 29663; 29611; 29610; 29605, 29645; 29612; 29602; 29662; 29642; 29641.
   b. Diagnoses for mental health specific hospitalizations and emergency room visits: Mental health specific admissions were counted if they had a primary diagnosis with the following CCS code[7]: 650, 651, 652, 655, 656, 657, 658, 659, 662, 663, 670.

4. **Additional information on key variables:**

We fixed VBP at the first year of implementation for several reasons. First, a majority of practices begin VBP in this year (~70%) and the attrition rate of practices out of VBP is very low (<1%). Second, practices who join in later years have substantially less time to plan and execute VBP funded delivery reform projects and are, therefore, a less representative group for the effects of VBP. Third, by fixing treatment at baseline, we do not introduce additional selection bias of practices joining VBP after witnessing the benefits of the program. In Supplemental Section 8, we conduct sensitivity analyses with time-varying VBP indicators and samples to assess whether coding late-VBP entrants as non-VBP biases our main estimates.

5. **Regression model specifications**

Our main empirical strategy was a difference-in-differences (DiD) analysis. We stratify the analysis by psychiatric diagnosis and estimate the following model:

$$Y_{itk} = \beta_0 + \beta_1 VBP_k x Post_t + \alpha_t + \alpha_i + \varepsilon_{itk}$$

Where Y_{ikt} is the expected outcome for an individual patient *i* in a given year *t* attributed to practice k. α_t is year fixed effects, and α_i is patient-level fixed effects. The patient and year level fixed-effects control for time-invariant observable and unobservable factors at the patient and year level, including the individual indicators for the pre-VBP period and attribution to VBP. $VBP_k x Post_t$ is the interaction between the indicator for a patient attributed to a VBP practice and the indicator being in the post-VBP period. The coefficient β_1 is the coefficient of interest, and estimates the adjusted mean differential change in utilization due to a patient's practice participation in VBP incentives compared to the non-VBP group. We used a linear regression and cluster standard errors at the practice level (the level of treatment assignment).

For reference, we also include an unadjusted model that lacks patient and year level fixed effects in ETables 2 and 3. The unadjusted model is specified by the following model:

$$Y_{itk} = \beta_0 + \beta_1 VBP_k + \beta_2 Post_t + \beta_3 VBP_k x Post_t + \varepsilon_{itk}$$

Where Y_{ikt} is the expected outcome for an individual patient *i* in a given year *t* attributed to provider k. VBP_k indicates if a patient is attributed to VBP participating practice, and $Post_t$ is an indicator for being in the post-VBP implementation period. The interaction term between $VBP_k x Post$

© 2023 Lewis A et al. *JAMA Health Forum*.

represents the differential change in outcomes for a patient whose outpatient practice participates in VBP reform compared to the non-VBP group in the post-VBP period.

6. Construction of utilization outcomes in the Medicaid claims data

Outcomes are the number of visits per patient-year. Visits are identified as an aggregation of claims per patient-NPI-date so that all claims associated with one visit are counted as a single visit. To categorize visits, we use a Category of Service variable derived from an algorithm of NPI specialty codes, place of service variables, procedure codes, and rate codes to classify claims as belonging to a service (primary care vs mental health, etc).

7. Comparing patient characteristics between analytic sample and those excluded due to insufficient enrollment.

By restricting the analytic population to Medicaid patients with 12 months of continuous enrollment, we may be selecting a particular patient population that is different from the general Medicaid population and whose underlying characteristics confound our measured associations. We compared patient-level characteristics of the analytic population and those excluded due to insufficient enrollment to evaluate potential underlying differences. The analytic population has 12 months of enrollment starting in July 1 of each year. The insufficient enrollment population are all patients who do not meet this criterion in a given year. Overall, the characteristics between the analytic population and the population excluded because of insufficient enrollment are similar across key characteristics. There are 1,486,229 number of patient-years excluded throughout the study period. Supplemental ETable 2 compares characteristics between analytic and excluded populations. The analytic and excluded populations are comparable for average age (40.8 vs 40.5 years, respectively), sex (64% female vs 60% female, respectively), and residence in NYC (50% vs 50%, respectively). There is relative balance across ethnicity between analytic population and excluded populations [non-Hispanic White: 39% v 44%; Black: 20% vs 17%; Asian: 4.0% vs 4.0%; Native American: 4% vs 5%; non-white Hispanic: 18% vs 14%]. The analytic population vs excluded population is slightly more likely to be attributed to the VBP group (80% vs 75%), has a slightly higher average Charlson co-morbidity index (2.1 vs 1.6), and slightly higher managed care enrollment (95.3% vs 90.9%). Our analytic population has higher total enrollment in the study period (70.1 months vs 44.9 months), which aligns with the exclusion criterion (insufficient enrollment).

8. Robustness Checks

All-cause hospitalizations and emergency room visits

Patients with mental illness have a higher burden of physical chronic illnesses, and tend to have higher proportion of adverse health events such as hospitalizations and emergency room visits compared to the general population. Previous studies on mental health interventions use all-cause hospitalizations and emergency room visits as proxies for the quality-of-care mental health patients receive related to their chronic disease management.[8, 9] In addition to mental-health related hospitalizations and emergency room visits, we also examined VBPs impact on all-cause admissions (Supplemental ETable 3).

We find that for patients in every diagnosis, VBP was associated with significant reductions in all-cause emergency room visits (Depression: -0.08 [-0.14,-0.02]; Bipolar: -0.15 [-0.27,-0.04]; Schizophrenia: -0.21, [-0.37,-0.05]) and that patients with depression have significant reductions in all-cause hospitalization (-0.02, [-0.03, -0.004]). This suggests that the population with mental illness also benefit from VBP reform for their physical illness management.

Time-varying VBP indicator

We explore how time-varying NPI participation in VBP may influence the association VBP has with our outcomes. First, we construct a VBP indicator which is 1 for every year an NPI is in our VBP dataset and zero when they are absent. 8.1% of patient-years become treated if we allow their attributed practice to enter VBP after the baseline year. We then performed a difference-in-differences analysis

© 2023 Lewis A et al. *JAMA Health Forum*.

using the adjusted model described in Supplemental eSection 5 using this time-varying VBP indicator. The interaction term between the VBP indicator (time-varied throughout the post-period) and post-VBP indicator will be the average, differential effect of VBP on outcomes after VBP is implemented. Results are presented in Supplemental eTable 4 and Supplemental eTable 5. Overall, we find that our results remain similar in both magnitude, direction, and precision to our main specification. Next, we exclude patients whose attributed practice is non-VBP at baseline but then enters VBP in the post-period. This allows us to measure if and to what degree our main specification estimates could be biased due to misclassification of late-entering VBP practices as non-VBPs. On the new analytic population of we perform a difference-in-differences using the adjusted model described in Supplemental Section 5 with the results in Supplemental eTable 6 and Supplemental eTable 7. Overall, we find that our results remain similar in both magnitude, direction, and precision to our main specification. The combined results of the time-varying VBP indictor analyses suggest that changes throughout the post-period in practice-level VBP status do not significantly drive our main estimations.

Inclusion of pre-VBP outcome trends into model

In the results of Figure 2, we detect small but significant pre-trends for the primary care outcome. To test the parallel trends assumption, we estimated our main model with an additional covariate to test for differences between VBP and non-VBP groups in pre-intervention trends in outcomes. This term was specified as an interaction between VBP_k and a linear time trend (indicator for Pre-VBP period), which estimates the differential yearly change in the outcome prior to VBP implementation and after VBP implementation in VBP patients versus the non-VBP patients. Accepting the null hypothesis would be consistent with the parallel trends assumption. Our results are in Supplemental eTable 8 and Supplemental eTable 9. The results for our primary outcomes remain relatively similar. The estimates for the linear pre-trend, which estimates if a differential change during the pre-VBP period is detected in the VBP group relative to the non-VBP, is significant for the primary care outcome trend for bipolar and schizophrenia VBP patients. No other outcomes demonstrate a significant differential trend during the pre-VBP period. Our estimates measuring the differential change in primary care visits for VBP patients after VBP reform – the estimate of the association between VBP and primary care visits- demonstrate a statistically significant reduction in schizophrenia patients (-1.49, [-2.27, -0.27]), similar to our main findings, indicating pre-VBP outcome trends do not drive our results. For the remaining outcomes, mental health visits are significantly associated with VBP for depression and bipolar populations (Depression: 0.96, [0.55,1.38]; Bipolar: 1.06, [0.24,1.8]). This is similar to our main results. Emergency room visits remain statistically significantly associated with VBP for depression and bipolar patients, but not schizophrenia patients (Depression: -0.09, [-0.15,-0.03; Bipolar: -0.13, [-0.26,-0.01]; Schizophrenia: -0.18, [-0.35,0.00]). The effects for hospitalizations are attenuated with the inclusion of pre-VBP outcome trends, despite non-significant differential pre-VBP outcome trends.

Analysis of outcomes at the six-month period level

In order to assess the outcome trends and changes with more granularity, we visualized the outcome trends for the VBP and non-VBP patient groups at the six-month period level. This allows four periods pre-VBP and eight periods post-VBP. eFigure 1 depicts these outcome trends, stratified by diagnostic group. We find that our outcome trends appear to follow a generally parallel pattern across outcomes and diagnostic populations with the exception of mental health hospitalizations for patients with schizophrenia and bipolar disorder. The smaller patient population, rare event of mental health hospitalizations, and more granular time period likely leads to higher levels of noise. To assess whether, at the six-month interval, pre-trend deviations bias results, we conduct two difference-in-differences at the six-month period level: 1) estimating our main adjusted model (outlined in eSection 5) to measure the differential change in outcomes for VBP patients after VBP at the six-month period level and 2) estimating our model outlined in "*Inclusion of pre-VBP outcome trends into model*" that incorporated pre-trends into our model. The addition of pre-trends as a covariate will control for pre-trend deviations between the VBP and non-VBP group. Results for our main model specifications at the six-month level

© 2023 Lewis A et al. *JAMA Health Forum*.

are in eTable 10 and the results for the analysis with included pre-trends is in eTable 11. We find that for outpatient utilization, the direction and significance at the six-month level is similar to that of the year-level across diagnostic group. At the six-month level, we find that mental health hospitalizations results remain similar to the year level in significance and direction, but we find the effects on mental health ED visits attenuated.

Population Compositional changes: Patient characteristics as outcomes

The composition of patient characteristics between VBP and non-VBP patients could change over time. This is a concern if VBP or non-VBP providers are selecting patients according to their participation in VBP after implementation. To test whether patient characteristics change differentially for the VBP and non-VBP groups before and after VBP, we use age, sex, and patient co-morbidity as outcomes. These characteristics should be unrelated to VBP implementation and should not differentially change over time. Accepting the null hypothesis would represent a failure to detect of selection, along these characteristics. Patient age, sex, and Charlson co-morbidity score were set as dependent variables. Charlson co-morbidity is fixed at the pre-VBP score. Since these are fixed characteristics, patient fixed effects would produce total collinearity. Instead, we specify the following model:

$$Y_{itk} = \beta_0 + \beta_2 Year_t + \beta_3 VBP_k x Year_t + \alpha_t + \alpha_k + X_i + \varepsilon_{itk}$$

Where Y_{ikt} is a patient characteristic (age, sex, Charlson Co-morbidty) for an individual patient *i* in a given year *t* attributed to provider *k*. VBP_k indicates if a patient is attributed to VBP participating provider, $Year_t$ is an indicator for being in the post-intervention period, α_t is year fixed effects, $\alpha_k$is NPI fixed-effects, and X_i is the patient characteristics that are not the dependent variable (e.g. inclusion of age and sex when Charlson co-morbidity score is the dependent variable). The main effect of interest, the interaction between $VBP_k x Year_t$, is interpreted as the differential change in patient characteristic for the VBP vs non-VBP group in that year relative to the first year of our study period (July 1, 2013- July 1, 2014 and first pre-VBP year). Supplemental ETable 12 reports the estimates from this analysis.

Overall, we do not detect differential change in age, sex, and Charlson co-morbidity scores for VBP vs non-VBP groups throughout the course of the study. We do detect a small, statistically significant differential decrease in age for the VBP group in the post-VBP for the population with depression relative to the first year of the study period. We also detect a small relative increase in Charlson co-morbidity scores for the bipolar population in early years that is no longer statistically significant in the later study period years. This suggest there may be compositional changes in the VBP group over time. We test the extent to which these changes may drive our results by reducing our analytic sample to a balance panel with continuously enrolled patients (Supplemental Section 8: *Balanced Panel*)

Log-linear regression

Patients with mental illness have a wide range of utilization and studies on utilization can be biased due to extreme values or outliers. To mitigate the effects of utilization outliers on our estimated effect, we took the natural logarithm of each outcome and performed a log-linear model. We used these transformed outcomes as the dependent variables using the adjusted patient and year level fixed effects described in Supplemental Section 3. For observations where the outcome is zero, the zero was replaced with 0.1. The reported estimates and confidence intervals are transformed (exponentiated the coefficient and subtract 1) to reflect the relative percent change in outcomes. Supplemental eTable 13 and Supplemental eTable 14 report the estimates from this analysis. We find that our primary outcomes of mental health visits and primary care visits remain robust. In fact, we find that VBP is statistically significantly associated with reductions in the percent of primary care visits for depression and bipolar patients (Depression: -3.0%; [-5.0,-1.0]; Bipolar: -5.0%; [-9.0,-2.0]) and VBP is statistically significantly associated with mental health visits for schizophrenia patients (3.0%; [0.3, 7.0]). This indicates outliers may have suppressed the effects in these outcomes. For secondary outcomes, we find that the associations

© 2023 Lewis A et al. *JAMA Health Forum*.

are directionally similar to the main findings but the precision of the effects differ and the significance is attenuated.

Two-part model: Association of VBP on the change in any utilization per outcome and the change in utilization intensity per outcome

The main analysis estimates reflect aggregated utilization, but do not discern utilization changes related to changes along extensive margins (no utilization to any utilization, or vice versa) or the intensive margins (utilizers changing the intensity of their utilization). To distinguish the type of utilization changes after VBP reform, we conduct a two-part model. The model will first determine the likelihood of changing from zero to any utilization, then, conditional on non-zero utilization determine the degree of change in utilization. We performed a logistic regression which estimates whether VBP is associated with changes in a binary outcome, where zero represents no utilization in that outcome and one represents any utilization in that outcome. Next, conditional on non-zero utilization, we estimated a patient and year level fixed effects linear regression to assess the association between VBP and outcomes. The reported estimates of the logistic regression are the average marginal effects of the association of VBP in the post-VBP period and can be interpreted as the percent change in the likelihood of having any utilization. Supplemental ETable 15 and Supplemental ETable 16 report the estimates from these analyses. For depression and bipolar patients, we find that VBP is associated with an increase in the likelihood of any mental health visits (Depression: 31.0%, [20.4, 42.2]; Bipolar: 20%, [2.3, 37.7]) and, conditional on any mental health visits, an increase in the number of visits (Depression: 1.57 visits, [0.57, 2.57]; Bipolar: 1.74 visits, [0.27, 3.21]).

Notably for each diagnosis, we find a statistically significant reduction in the likelihood of any primary care utilization (Depression: -20.0%, [-32.1,-7.5]; Bipolar: -28.0%, [-43.8,-12.0]; Schizophrenia: -27.0%, [-45.1,-9.2]) but only schizophrenia has a statistically significant reduction in the intensity of primary care usage associated with VBP, conditional on any primary care utilization (-1.50 visits, [-2.89,-0.11]).

VBP was associated with a reduction in the likelihood of any ED visits only for depression patients, by 12.0% points (-0.18,-0.05). However, conditional on non-zero emergency room utilization, VBP was associated with a reduction in the number of emergency room visits for all diagnoses (Depression: -0.28, [-0.43,-0.13]; Bipolar: -0.42, [-0.68,-0.16]; Schizophrenia: -0.52 , [-0.92,-0.11]). The VBP association with hospitalizations for depression patients appears to be driven by a reduction in the probability in any hospitalizations, by 5.0% points (-9.0,-1.0).

Balanced Panel: Continuously enrolled population

Medicaid patients have significant enrollment churn. Our main analysis allows patient-year observations that meet the enrollment requirements to be included in the analysis, while excluding patient-year observations that fail to meet 12 months enrollment in that year. A potential threat to the analysis is that the patients disenrolling in Medicaid after VBP implementation are different from those disenrolled before VBP implementation, differential to VBP attribution in ways that confounds the estimated VBP association with our outcomes. In Supplemental ETable 12, we find that for VBP depression patients, there is a differential decrease in the age over time. This suggests the composition in the VBP depression group, along the dimension of age, could change differentially and confound our results. By restricting our analysis to a balanced panel, which requires beneficiaries to be enrolled for 12 months in every year of our study, we no longer have patients dis-enrolling in the study period. Therefore, the population characteristics are static in the study period. We restrict the population to patients who are continuously enrolled from July, 1 2013- July 1, 2019. On this balanced panel, we perform the same model described in Supplemental Section 5a.

3,384,912 (45.1%) patients were excluded from the balanced panel due to insufficient enrollment (not being continuously enrolled). Supplemental eTable 17 and Supplemental eTable 18 report the estimates from this analysis. We find consistent results with our main specification for behavioral health and primary care outcomes. VBP is statistically significantly associated with increased behavioral health

© 2023 Lewis A et al. *JAMA Health Forum*.

visits in depression and bipolar patients and with reductions in primary care for schizophrenia patients. For secondary outcomes, the associations with reductions in emergency room visits remain statistically significant and are of a similar magnitude to the main analysis (eTable 3). The VBP associations for hospitalization outcomes are a similar direction but the precision is attenuated. Overall, the restriction to a balanced panel demonstrates that differential composition changes in our population are not a driver of our findings.

Reduced analytic Population to patients with Consistent Exposure Status throughout post-VBP period

Medicaid patients have fragmented care, often seeing more than one provider or practice. This may interrupt consistent exposure to VBP reform, since patient may change providers or practices over time who may or may not be participating in DSRIP. To test how continuity to VBP exposure impacts our estimated associations, we restrict our sample to patients who, upon reattribution in the post-VBP period, do not switch treatment assignment from baseline their baseline status (i.e. VBP or non-VBP throughout the study period). Reattribution in the post-VBP period follows the same procedure outlined in Supplemental eSection 2 for the post-VBP years of July 1, 2016-July 1, 2017 and July 1, 2018-July 1, 2019. If the patient's attributed practice in both post-VBP years is that same group as their baseline practice (either in VBP or not in VBP), then we include this patient into the new population for this analysis. This procedure allows patients to be included in the new population even if their attributed practice changes, as long as the exposure status (VBP or non-VBP) is constant.

The model is the same as the one estimated in Supplemental Section 5. Supplemental ETable 19 and Supplemental ETable 20 report the estimated associations. We find that VBP's association with behavioral health visits are similar in statistical significance and magnitude as our main results. In contrast, for each diagnosis, we find that the association of VBP with primary care is statistically significant and in the inverse direction from the main results. Now VBP is associated with an increase in primary care visits rather than a decrease (Depression: 1.58, [1.0, 2.1]; Bipolar: 2 .62 [1.7, 3.5]; Schizophrenia: 1.79 [0.9, 2.7]). Our secondary outcomes- hospitalizations and emergency room visits- are not statistically significant or are very small (e.g., Depression, Mental Health Hospitalizations: -0.01, [0.0, 0.0]). For this higher-continuity population, the inverse direction of the association between VBP and primary care suggest that this utilization outcome may require longer-term exposure to VBP in order to translate into substantial changes.

Quality outcomes after VBP reform

Direct measures of quality improvements, such as depression screening information, are not available in administrative data.[10] However, ED visits and hospitalizations are highly correlated with healthcare quality and have been used has valid proxies in claims data.[11, 12] Mental health related emergency room and hospitalizations are considered important measures of quality for the patient populations with mental illness, specifically. To further illustrate quality changes in DSRIP, we have included hospital readmissions and preventative hospitalizations as additional outcomes. Both of these outcomes were specific metrics used in pay-for-performance evaluations in DSRIP.[13] Readmissions were calculated as another inpatient hospital admission (all-cause) within 30 days of an inpatient admission (all-cause). Preventable hospitalizations were defined as any admission with a primary diagnosis that met the requirements for preventable admission in AHRQ's PQI #92 Chronic Disease Indicator.

Supplement eTable 21 demonstrates the results of the difference-in-difference analysis for 30-day readmissions and preventable hospitalizations. Overall, we find no significant changes in these outcomes associated with VBP reform. This may suggest that the VBP reform for mental health patients may have had the most impact directly on mental healthcare rather than more general health quality. This is noted by participating health systems and clinicians, which stated patients with mental illness were the population who most benefited from reform and most noted mental healthcare was positively transformed.[14]

© 2023 Lewis A et al. *JAMA Health Forum*.

Combined outpatient utilization after VBP reform

In our main analysis, we find VBP is associated with an increase in mental health visits for depression and bipolar patients and a reduction in primary care for schizophrenia patients. We investigate whether there is a net change in overall outpatient visits to investigate whether there is a net change in outpatient usage, by combining primary care and mental health visits together. Supplemental ETable 22 reports the estimates from this analysis. Overall, we are finding that there is not a significant net change in outpatient utilization, suggesting that there is a redistribution of the outpatient services.

Mental Health-related Primary care visits after VBP reform

Primary care is an important delivery site for mental healthcare. A potential consequence of shifting the increased mental health visits we observe is that less mental health is delivered in the primary setting creating an unintended barrier to mental health services. To test whether this is the case in our population, we perform an exploratory analysis on the changes to mental-health related primary care visits after VBP was enacted. We identified mental-health related primary care visits (MHPC) by identifying visits with ICD procedure codes matching psychiatric or psychological related services (ICD 9: 9401-9409, 9411-9419, 9421-9429, 9431-939, 9441-9449, 9451-9459, 9461-9469; ICD 10: 'GZ10ZZZ','GZ11ZZZ','GZ12ZZZ','GZ13ZZZ','GZ14ZZZ','GZ2ZZZZ','GZ3ZZZZ','GZ50ZZZ','GZ51ZZZ','GZ52ZZZ','GZ53ZZZ','GZ54ZZZ','GZ55ZZZ','GZ56ZZZ','GZ58ZZZ','GZ59ZZZ','GZ60ZZZ','GZ61ZZZ','GZ63ZZZ','GZ72ZZZ','GZB0ZZZ','GZB1ZZZ','GZB2ZZZ','GZB3ZZZ','GZB4ZZZ','GZC9ZZZ','GZFZZZZ','GZGZZZZ','GZHZZZZ','GZJZZZZ').

In administrative claims data, it is difficult to measure exact services delivered due to coding inconsistencies with mental healthcare, often biasing toward under-reporting.[15, 16] Therefore, this outcome likely underestimates the actual prevalence of mental health services provided during primary care visits. Supplemental ETable 23 reports the estimates from this analysis. We find that, compared to control patients, VBP patients with depression and bipolar disorder have a relative increase in MHPC visits after reform is implemented [Depression: 0.02 visits (0.01, 0.04); Bipolar: 0.02, (0.0031, 0.04)], while patients with schizophrenia have no significant difference. Although preliminary, this analysis does not provide evidence that VBP is differentially impeding access to mental healthcare in primary care settings.

Multiple Comparisons Correction

Because we use multiple diagnostic populations and utilization outcomes, our results could be at risk of detecting a significant result that is not true (Type 1 error). To account for this, we corrected our adjusted regression results by applying the false discovery rate procedure (Benjamini and Yekutieli method),[17] which allows for correlation across tests. This procedure adjusts the p-values for each estimate and calculates a new significance level to reject the null hypothesis.

Supplemental eTable 24 depicts the results from the multiple testing analysis with corrected p-values and the adjusted significance value of 0.021, meaning if the adjusted p-values are below 0.021 then the null hypothesis (VBP is not associated with a change in outcome) can be rejected. We find that for patients with depression, VBP was significantly associated with increased mental health visits and decreased mental health hospitalizations and mental health ED. For patients with bipolar disorder, VBP are associated with increased mental health visits. Outpatient utilization and mental health ED and hospitalizations changes were not significantly associated with VBP for patients with schizophrenia

9. Heterogeneity Analysis

Comparison of effects for patients in New York City vs Rest of New York state

There was significant geographic heterogeneity on the focus of VBP projects and target populations for NY DSRIP and there is significant variation in the Medicaid populations across the state. The largest contrast is between New York City (NYC) versus non-NYC DSRIP programs. NYC's Medicaid

population generally has higher disease burden, more likely to be non-White, qualify based on SSI, and older than those in the rest of state.[18, 19] The NYC DSRIP networks typically had a smaller geographical capture area which had overlap with other DSRIP networks while non-NYC DSRIP networks spanned several counties and sometimes were the only DSRIP networks in the county. NYC DSRIP networks had a larger average number of engaged mental health providers in the network as well (296 vs 148 mental health providers in collaborative care). We aim to assess how the VBP-outcome associations may vary according to these geographical differences. We created an indicator for if a patient's zip code was a NYC zipcode. This zip-code was set at baseline (July 1-2014 – June 30, 2015). We then performed the adjusted patient and year level fixed effects model described in Supplemental Section 5, stratified by patient zip-code.

Supplemental ETable 25 and Supplemental ETable 26 report the results from this analysis. We find that VBP is associated with statistically significant reduction in primary care across diagnoses for non-NYC VBP patients (Depression: -0.91, [-1.2,-0.6]; Bipolar: -1.51, [-2.0,-1.0]; Schizophrenia: -1.74; [-2.4,-1.1]) but not NYC VBP patients. Overall, VBP is statistically significantly associated with mental health increases for both NYC and non-NYC. VBP associated reductions in emergency room visits appear to be driven by non-NYC VBP patients whereas NYC patients have no significant changes in hospitalizations or emergency room visits, except a very small reduction in mental health hospitalizations (-0.01, [0.0,0.0]).

Stratification by co-morbidity burden in schizophrenia patients

We found VBP was associated with significant and large reductions in primary care (Table 2) and ED visits (Supplemental ETable 1a) for schizophrenia patients. This result counters our hypothesis that outpatient utilization (primary care) is inversely related to hospitalization and emergency room utilization. Previous literature has shown that Medicaid schizophrenia patients have different utilization in primary care and emergency room visits based on the severity of co-morbidity burden. Medicaid schizophrenia patients with more co-morbidities have higher utilization, generally, in order to manage complex physical chronic illnesses.[20] Therefore, the VBP associations of reduced primary care and emergency room visits may be happening in different disease-burdened populations. To investigate whether VBP has heterogeneous impact for the schizophrenia population based on co-morbidity burden, we stratify our model (Supplemental eSection 5) by a categorical Charlson Co-Morbidity score. Lower scores represent fewer co-morbidities. Supplemental ETable 27 reports the stratified associations. We find that the means of our outcomes, except for mental health hospitalizations and mental health ED visits, increase as co-morbidity score increases. This aligns with the higher utilization driven by physical illness rather than increased severity of mental illness. We find that VBP is statistically significantly associated with primary care reduction (-1.31, [-2.1, -0.49]) only in patients with the lowest co-morbidity burden. We also find that VBP is only statistically significantly associated with emergency room reductions (-0.56, [-1.06, -0.06]) in patients with the highest co-morbidity burden. This demonstrates that VBP associated primary care reductions are concentrated in patients with the fewest co-morbidities, who likely have less chronic illness management. This may indicate fewer primary care visits is clinically appropriate. For patients with the highest degree of need, we find not changes in primary care but do find VBP associated emergency room reductions, which was a core aim of NY DSRIP program.

© 2023 Lewis A et al. *JAMA Health Forum*.

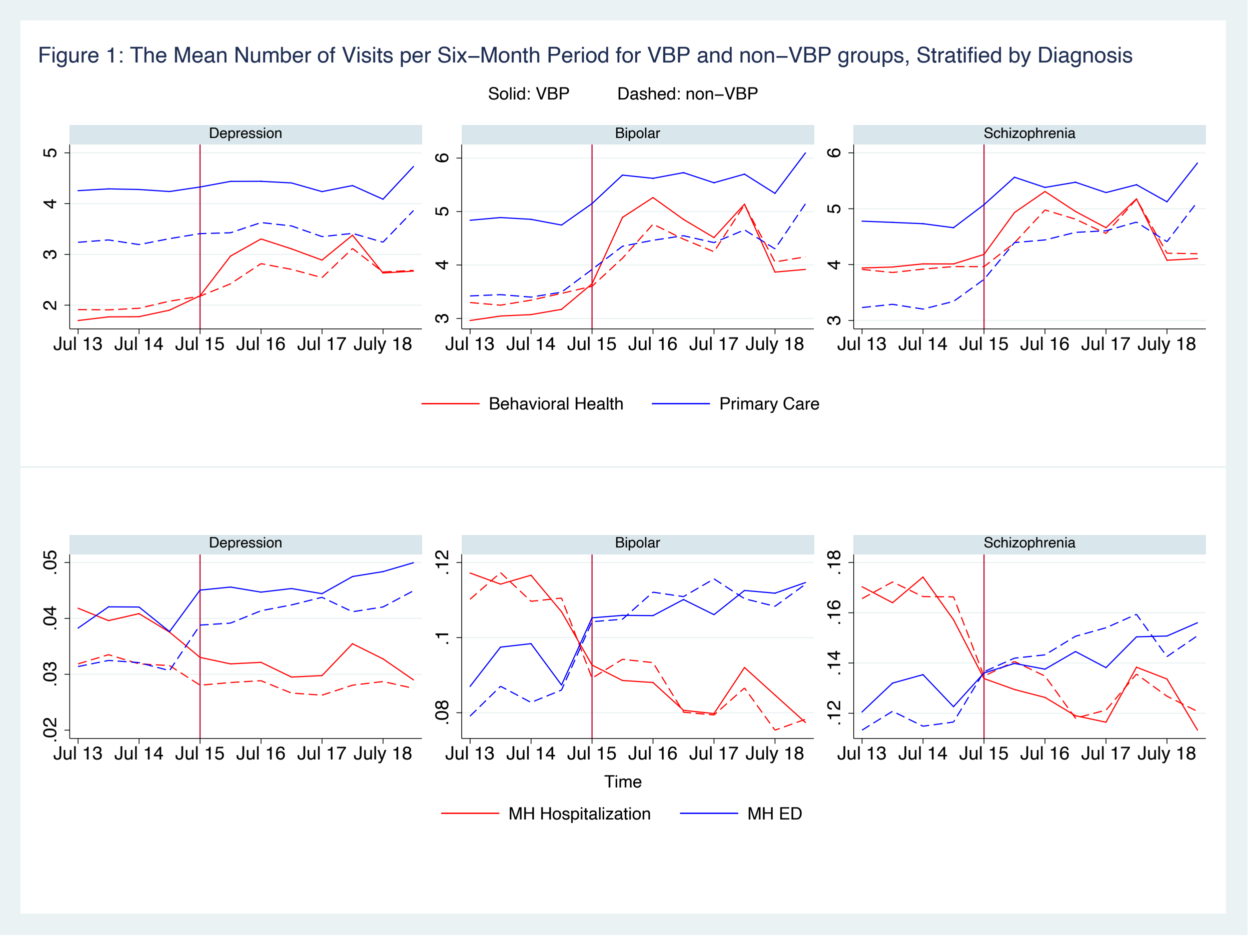

eFigure. The Mean Number of Visits of six-month period for VBP and non-VBP groups, stratified by diagnosis. The y-axis represents the means patient utilization by VBP status throughout our study period (x-axis). The red vertical line indicates VBP implementation on July 1, 2015. "MH" refers to "mental health".

© 2023 Lewis A et al. JAMA Health Forum.

eTable 1. Characteristics of NPIs in analytic sample by VBP participation and NPI type

	VBP NPIs		non-VBP NPIs	
	Individual	Organization	Individual	Organization
Total (N, %)[a]	16,752 (94.1%)	1,051 (5.9%)	7,719 (93%)	582 (7%)
Average Panel Size[b]	41	1545	35	392
% PCP	66.5	4.8	55.8	4.1
% Specialty	33.5	95.2	44.2	95.9
% Safety-Net[c]	32.1	10.8	15.7	8.4

a. Total of unique NPIs in analytic samples
b. Average number of unique patients per NPI
c. Percent of NPI-type that is safety-net as indicated on NY DOH website

© 2023 Lewis A et al. *JAMA Health Forum*.

eTable 2. Pre-VBP Characteristics of Analytic Population vs. Patients Excluded for Insufficient Enrollment

	Analytic	Excluded
Total (N)	1,875,948	1,486,229
Age (mean)	40.8	40.5
Female (%)	64.0	60.0
Race/ethnicity (%)		
White	38.9	43.5
Black	19.8	16.7
Asian	4.0	3.9
Native American	4.4	4.6
Hispanic	18.4	13.5
Missing	14.6	17.7
Total Months Enrolled (study period)	70.1	44.9
Managed Care (%)	95.3	90.9
Charlson comorbidity Index (mean)	2.1	1.6
NYC (%)	47.8	47.2
Attributed to VBP (%)	80.2	74.8

© 2023 Lewis A et al. *JAMA Health Forum*.

eTable 3. Differential changes in all-cause hospitalizations and ED visits for VBP patients after VBP

	VBP		non-VBP		Adjusted	
	Before VBP	After VBP	Before VBP	After VBP		
**Depression**						
Number of Visits						
Hospitalization	0.43	0.37	0.38	0.33	-0.02<br>[-0.03,-0.004]	0.01
ED	0.95	1.39	0.83	1.35	-0.08<br>[-0.14,-0.02]	0.01
**Bipolar**						
Number of Visits						
Hospitalization	0.71	0.58	0.68	0.57	-0.01<br>[-0.04,0.01]	0.30
ED	1.46	2.00	1.37	2.07	-0.15<br>[-0.27,-0.04]	0.01
**Schizophrenia**						
Number of Visits						
Hospitalization	0.86	0.67	0.87	0.69	-0.02<br>[-0.05,0.02]	0.41
ED	1.59	2.08	1.59	2.29	-0.21<br>[-0.37,-0.05]	0.01

© 2023 Lewis A et al. *JAMA Health Forum*.

eTable 4. Time-varying VBP indicator throughout study period: Differential change in primary outcomes after VBP for VBP patients

		Estimates (95% CI)	p-value
Depression			
Number of Visits			
	Behavioral Health	0.94 [0.51,1.36]	<0.001
	Primary Care	-0.54 [-1.08,-0.01]	0.05
Bipolar Depression			
Number of Visits			
	Behavioral Health	1.07 [0.21,1.92]	0.02
	Primary Care	-1.05 [-2.05,-0.05]	0.04
Schizophrenia			
Number of Visits			
	Behavioral Health	0.4 [-0.66,1.46]	0.46
	Primary Care	-1.66 [-2.82,-0.50]	0.005

© 2023 Lewis A et al. *JAMA Health Forum*.

eTable 5. Time-varying VBP indicator throughout study period: Differential change in secondary outcomes after VBP for VBP attributed patients

		Estimates (95% CI)	p-value
Depression			
Number of Visits			
	Hospitalization	-0.01 [-0.03,-0.00]	0.02
	Mental Health Hospitalizations	-0.01 [-0.01,-0.00]	<0.001
	ED	-0.08 [-0.14,-0.02]	0.008
	Mental Health ED	-0.01 [-0.01,-0.00]	0.04
Bipolar Depression			
Number of Visits			
	Hospitalization	-0.01 [-0.04,0.02]	0.51
	Mental Health Hospitalizations	0 [-0.02,0.01]	0.63
	ED	-0.13 [-0.24,-0.03]	0.02
	Mental Health ED	-0.02 [-0.04,0.00]	0.10
Schizophrenia			
Number of Visits			
	Hospitalization	-0.01 [-0.05,0.03]	0.70
	Mental Health Hospitalizations	0 [-0.02,0.02]	0.87
	ED	-0.21 [-0.37,-0.06]	0.007
	Mental Health ED	-0.03 [-0.05,0.00]	0.08

© 2023 Lewis A et al. *JAMA Health Forum*.

eTable 6. Restricted to patients whose practice enters VBP in the First VBP year: Differential change in primary outcomes after VBP for baseline VBP patients

		Estimates (95% CI)	p-value
Depression			
Number of Visits			
	Behavioral Health	0.98 [0.54,1.41]	<0.001
	Primary Care	-0.58 [-1.13,-0.02]	0.04
Bipolar Depression			
Number of Visits			
	Behavioral Health	1.11 [0.24,1.97]	0.01
	Primary Care	-1.08 [-2.12,-0.04]	0.04
Schizophrenia			
Number of Visits			
	Behavioral Health	0.4 [-0.67,1.48]	0.46
	Primary Care	-1.65 [-2.87,-0.43]	0.008

© 2023 Lewis A et al. *JAMA Health Forum*.

eTable 7. Restricted to patients whose practice enters VBP in the First VBP year: Differential change in secondary outcomes after VBP for baseline VBP patients

		Estimates (95% CI)	p-value
Depression			
Number of Visits			
	Hospitalization	-0.01 [-0.03, 0.00]	0.02
	Mental Health Hospitalizations	-0.01 [-0.01, 0.00]	<0.001
	ED	-0.07 [-0.13, -0.01]	0.02
	Mental Health ED	-0.01 [-0.01, 0.00]	0.04
Bipolar Depression			
Number of Visits			
	Hospitalization	-0.01 [-0.04,0.02]	0.42
	Mental Health Hospitalizations	0 [-0.02,0.01]	0.52
	ED	-0.12 [-0.24,-0.01]	0.03
	Mental Health ED	-0.02 [-0.04,0.00]	0.12
Schizophrenia			
Number of Visits			
	Hospitalization	-0.02 [-0.05,0.02]	0.43
	Mental Health Hospitalizations	-0.01 [-0.03,0.01]	0.51
	ED	-0.2 [-0.36,-0.04]	0.02
	Mental Health ED	-0.03 [-0.06,0.00]	0.07

© 2023 Lewis A et al. *JAMA Health Forum*.

eTable 8. Incorporation of Pre-VBP outcome trends: Differential change in primary outcomes after VBP for VBP patients

		Differential change in outcome trends for VBP patients in the pre-VBP period		Differential change in outcome trend for VBP patients in the post-VBP period	
		Estimates (95% CI)	p-value	Estimates (95% CI)	p-value
Depression					
Number of Visits					
	Behavioral health	0.1 [-0.04,0.25]	0.16	0.96 [0.55,1.38]	<0.001
	Primary care	-0.14 [-0.31,0.03]	0.11	-0.44 [-0.99,0.11]	0.12
Bipolar					
Number of Visits					
	Behavioral health	0.1 [-0.22,0.41]	0.54	1.06 [0.24,1.8]	0.01
	Primary care	-0.34 [-0.63,-0.04]	0.03	-0.89 [-1.91,0.12]	0.08
Schizophrenia					
Number of Visits					
	Behavioral health	0.07 [-0.38,0.53]	0.75	0.3 [-0.74,1.33]	0.58
	Primary care	-0.35 [-0.63,-0.06]	0.02	-1.49 [-2.72,-0.27]	0.02

© 2023 Lewis A et al. *JAMA Health Forum*.

eTable 9. Incorporation of Linear Pre-VBP outcome trends: Differential change in secondary outcomes after VBP for VBP patients

	Differential change in outcome trend for VBP patients in the pre-VBP period		Differential change in outcome trend for VBP patients in the post-VBP period	
	Estimates (95% CI)	p-value	Estimates (95% CI)	p-value
Depression				
Number of Visits				
Hospitalization	0 [-0.01,0.01]	0.82	-0.01 [-0.03,-0.001]	0.03
Mental Health Hospitalizations	0 [-0.01,0.002]	0.28	-0.01 [-0.02,-0.004]	<0.001
ED	-0.01 [-0.04,0.02]	0.42	-0.09 [-0.15,-0.03]	0.01
Mental Health ED	0 [-0.005,0.01]	0.77	-0.01 [-0.02,-0.001]	0.04
Bipolar Depression				
Number of Visits				
Hospitalization	0 [-0.03,0.03]	0.87	-0.02 [-0.05,0.02]	0.37
Mental Health Hospitalizations	-0.01 [-0.02,0.01]	0.52	-0.01 [-0.03,0.01]	0.35
ED	0.04 [-0.03,0.11]	0.24	-0.13 [-0.26,-0.01]	0.04
Mental Health ED	0 [-0.02,0.01]	0.69	-0.02 [-0.05,0.00]	0.05
Schizophrenia				
Number of Visits				
Hospitalization	0.02 [-0.02,0.06]	0.35	-0.01 [-0.05,0.04]	0.81
Mental Health Hospitalizations	0 [-0.03,0.02]	0.67	-0.01 [-0.03,0.02]	0.45
ED	0.06 [-0.05,0.18]	0.26	-0.18 [-0.35,0.00]	0.05
Mental Health ED	0.01 [-0.02,0.03]	0.59	-0.03 [-0.07,0.00]	0.05

© 2023 Lewis A et al. *JAMA Health Forum*.

eTable 10. Differential change in outcomes for VBP patients after VBP at the six-month period level

	VBP		non-VBP		Adjusted^{b}	
	Before VBP	After VBP	Before VBP	After VBP		
**Depression**					Estimates (95% CI)	p-value
Number of Visits per six months						
Behavioral Health	1.8	2.9	2.0	2.6	0.45 [0.2573,0.6502]	<0.001
Primary care	4.3	4.4	3.3	3.5	-0.18 [-0.4459,0.0815]	0.176
Mental Health Hospitalizations	0.04	0.031	0.032	0.028	-.004 [-0.01,-0.002]	<0.001
Mental Health ED	0.04	0.047	0.032	0.42	-.004 [-0.01,-0.001]	0.01
**Bipolar**						
Number of Visits per six months						
Behavioral Health	3.1	4.5	3.3	4.3	0.5 [0.1109,0.8963]	0.01
Primary care	4.8	5.6	3.4	4.5	-0.36 [-0.8522,0.1379]	0.16
Mental Health Hospitalizations	0.11	0.09	0.11	0.09	-0.01 [-0.02,0.001]	0.06
Mental Health ED	0.09	0.11	0.08	0.11	-0.01 [-0.03,0.003]	0.11
**Schizophrenia**						
Number of Visits per six months						
Behavioral Health	4.0	4.7	3.9	4.5	0.13 [-0.3610,0.6170]	0.61
Primary care	4.7	5.4	3.3	4.5	-0.66 [-1.2541,-0.0584]	0.03
Mental Health Hospitalizations	0.17	0.13	0.17	0.13	-0.01 [-0.03,0.004]	0.15
Mental Health ED	0.13	0.14	0.12	0.15	-0.01 [-0.03,0.005]	0.13

© 2023 Lewis A et al. *JAMA Health Forum*.

eTable 11. Incorporation of Pre-VBP outcome trends at the six-month period level: Differential change in primary outcomes after VBP for VBP patients

	Differential change in outcome trend for VBP patients in the post-VBP period	
	Estimates (95% CI)	p-value
Depression		
Number of Visits: per six months		
Behavioral Health	0.52 [0.31,0.73]	<0.001
Primary Care	-0.2259 [-0.5,0.05]	0.11
Mental Health Hospitalization	-0.01 [-0.01,-0.004]	<0.001
Mental Health ED	-0.003 [-0.01.002]	0.21
Bipolar Depression		
Number of Visits: per six months		
Behavioral Health	0.60 [0.18,1.01]	0.005
Primary Care	-0.43 [-0.93,0.07]	0.09
Mental Health Hospitalization	-0.009 [-0.02,0.001]	0.06
Mental Health ED	-0.01 [-0.023,0.003]	0.11
Schizophrenia		
Number of Visits: per six months		
Behavioral Health	0.18 [-0.36,0.73]	0.51
Primary Care	-0.79 [-1.38,-0.19]	0.01
Mental Health Hospitalization	-0.01 [-0.03,0.004]	0.15
Mental Health ED	-0.01 [-0.03,0.005]	0.13

© 2023 Lewis A et al. *JAMA Health Forum*.

eTable 12. Differential changes in patient characteristics for VBP vs non-VBP patients in each study year relative to July 2013-2014

		Pre-VBP	Post-VBP			
		Jul 2014-2015	Jul 2015-2016	Jul 2016-2017	Jul 2017-2018	Jul 2018-2019
		Estimates (95% CI)				
Depression						
	Age	-0.01 [-0.09,0.07]	-0.13 [-0.22,-0.04]	-0.13 [-0.23,-0.03]	-0.14 [-0.24,-0.04]	-0.22 [-0.33,-0.11]
	Gender	0 [0.00,0.00]	0 [0.00,0.00]	0 [0.00,0.01]	0 [-0.01,0.00]	0 [0.00,0.01]
	Co-morbidity Score	0.03 [0.01,0.05]	0.03 [0.00,0.05]	0.03 [0.00,0.06]	0.02 [-0.01,0.05]	0.01 [-0.03,0.04]
Bipolar						
	Age	0.08 [-0.04,0.21]	0.08 [-0.06,0.23]	0.07 [-0.09,0.23]	0.04 [-0.13,0.21]	0.05 [-0.14,0.23]
	Gender	0 [-0.01,0.01]	0 [-0.01,0.01]	0 [-0.01,0.01]	0 [-0.01,0.01]	0 [-0.01,0.01]
	Co-morbidity Score	0.05 [0.02,0.09]	0.05 [0.04,0.09]	0.02 [-0.03,0.07]	0.01 [-0.04,0.06]	0.02 [-0.04,0.05]
Schziophrenia						
	Age	0.06 [-0.09,0.22]	0.07 [-0.11,0.25]	0.04 [-0.15,0.22]	0.05 [-0.14,0.24]	0.03 [-0.17,0.23]
	Gender	0 [-0.01,0.00]	0 [-0.01,0.00]	-0.01 [-0.01,0.00]	0 [-0.01,0.01]	0 [-0.01,0.01]
	Co-morbidity Score	0.03 [-0.03,0.08]	0.04 [-0.02,0.10]	0.02 [-0.04,0.09]	0.02 [-0.06,0.10]	0.02 [-0.06,0.10]

© 2023 Lewis A et al. *JAMA Health Forum*.

eTable 13. Log-transformed primary outcomes: Differential change in outcomes after VBP for VBP attributed patients

		Relative % change in outcomes for VBP patients after VBP reform	
		Estimates (95% CI)	p-value
Depression			
Number of Visits			
	Behavioral Health	5.0 [2.0,7.0]	<0.001
	Primary Care	-3.0 [-5.0,-1.0]	0.009
Bipolar Depression			
Number of Visits			
	Behavioral Health	4.0 [1.0,7.0]	0.01
	Primary Care	-5.0 [-9.0,-2.0]	0.003
Schizophrenia			
Number of Visits			
	Behavioral Health	3.0 [0.3,7.0]	0.03
	Primary Care	-7.0 [-12.0,-3.0]	0.002

© 2023 Lewis A et al. *JAMA Health Forum*.

eTable 14. Log-transformed secondary outcomes: Differential change in outcomes after VBP for VBP attributed patients

		Relative % change in outcomes for VBP patients after VBP reform	
		Estimates (95% CI)	p-value
Depression			
Number of Visits			
	Hospitalization	-1.0 [-6.0, 5.0]	0.76
	Mental Health Hospitalizations	-14.0 [-26.0, 0.0]	0.05
	ED	-3.0 [-7.0, 0.30]	0.07
	Mental Health ED	10.0 [-9.0, 32.0]	0.33
Bipolar Depression			
Number of Visits			
	Hospitalization	-2.0 [-9.0, 5.0]	0.60
	Mental Health Hospitalizations	-12.0 [-25.0, 3.0]	0.10
	ED	-4.0 [-9.0, 1.0]	0.08
	Mental Health ED	5.0 [-14.0, 28.0]	0.62
Schizophrenia			
Number of Visits			
	Hospitalization	-3.0 [-10.0, 4.0]	0.40
	Mental Health Hospitalizations	-14.0 [-26.0, 1.0]	0.06
	ED	-6.0 [-12.0,-1.0]	0.03
	Mental Health ED	7.0 [-12.0, 31.0]	0.47

© 2023 Lewis A et al. *JAMA Health Forum*.

eTable 15. Two Part Model: Differential change in primary outcomes after VBP for VBP patients

		Percentage point change in differential probability of having any utilization for VBP patients compared to non-VBP patients after VBP reform		Change in outcomes for VBP patients after VBP reform, conditional on non-zero outcome	
		Estimates (95% CI)	p-value	Estimates (95% CI)	p-value
Depression					
Number of Visits					
	Behavioral health	3.5	<0.001	1.57	0.002
		[2.2, 4.8]		[0.57, 2.57]	
	Primary care	-1.02	0.03	-0.25	0.40
		[-1.9,-0.001]		[-0.84,0.33]	
Bipolar Depression					
Number of Visits					
	Behavioral health	2.6	0.02	1.74	0.02
		[0.004, 4.9]		[0.27, 3.21]	
	Primary care	-1.4	0.02	-0.66	0.25
		[-2.8,-0.002]		[-1.78,0.46]	
Schizophrenia					
Number of Visits					
	Behavioral health	0.01	0.77	0.49	0.52
		[-3.0, 4.0]		[-1.03, 2.02]	
	Primary care	-1.7	0.02	-1.5	0.03
		[-3.2,-0.002]		[-2.89,-0.11]	

© 2023 Lewis A et al. *JAMA Health Forum*.

eTable 16. Two Part Model: Differential change in secondary outcomes after VBP for VBP patients

	Percentage point change in differential probability of having any utilization for VBP patients compared to non-VBP patients after VBP reform		Change in outcomes for VBP patients after VBP reform, conditional on non-zero outcome	
	Estimates, % (95% CI)	p-value	Estimates (95% CI)	p-value
**Depression**				
Number of Visits				
Hospitalization	-1.0	<0.001	-0.03	0.25
	[-1.3,-0.005]		[-0.09,0.03]	
Mental Health Hospitalizations	-0.4	<0.001	-0.11	0.06
	[-1.0,-0.2]		[-0.22,0.00]	
ED	-1.4	0.01	-0.28	<0.001
	[-2.4,-0.2]		[-0.43,-0.13]	
Mental Health ED	-0.30	<0.001	-0.16	0.26
	[-0.5,-0.1]		[-0.43,0.12]	
**Bipolar**				
Number of Visits				
Hospitalization	-1.0	0.19	-0.06	0.25
	[-1.2, 0.2]		[-0.15,0.04]	
Mental Health Hospitalizations	-0.4	0.13	-0.01	0.80
	[-1.0,0.10]		[-0.12,0.09]	
ED	-1.0	0.39	-0.42	0.002
	[-2.0,1.0]		[-0.68,-0.16]	
Mental Health ED	-1.0	0.11	-0.18	0.37
	[-1.1,0.1]		[-0.57,0.21]	
**Schizophrenia**				
Number of Visits				
Hospitalization	-0.17	0.74	-0.07	0.17
	[-1.2,1.0]		[-0.18,0.03]	
Mental Health Hospitalizations	-0.44	0.23	-0.04	0.46
	[-1.2,0.3]		[-0.14,0.06]	
ED	-1.4	0.08	-0.52	0.01
	[-3.0,0.15]		[-0.92,-0.11]	
Mental Health ED	-0.90	0.06	-0.15	0.38
	[-1.7,-0.03]		[-0.48,0.18]	

© 2023 Lewis A et al. *JAMA Health Forum*.

eTable 17. Balanced Panel primary outcomes: Differential change after VBP for VBP attributed patients

		Estimates (95% CI)	p-value
**Depression**			
Number of Visits			
	Behavioral Health	1.01 [0.5,1.5]	<0.001
	Primary Care	-0.35 [-1.0,0.3]	0.32
**Bipolar**			
Number of Visits			
	Behavioral Health	1.07 [0.2,2.0]	0.02
	Primary Care	-0.82 [-2.0,0.4]	0.18
**Schizophrenia**			
Number of Visits			
	Behavioral Health	0.3 [-0.8,1.4]	0.60
	Primary Care	-1.58 [-3.0,-0.1]	0.03

© 2023 Lewis A et al. *JAMA Health Forum*.

eTable 18. Balanced Panel secondary outcomes: Differential change after VBP for VBP attributed patients

		Estimates (95% CI)	p-value
**Depression**			
Number of Visits			
	Hospitalization	-0.01 [-0.0,0.0]	0.09
	Mental Health Hospitalizations	-0.01 [-0.0,0.0]	0.01
	ED	-0.14 [-0.2,-0.1]	<0.001
	Mental Health ED	-0.01 [-0.0,0.0]	0.02
**Bipolar**			
Number of Visits			
	Hospitalization	-0.01 [0.0,0.0]	0.41
	Mental Health Hospitalizations	0 [0.0,0.0]	0.59
	ED	-0.15 [-0.3,0.0]	0.03
	Mental Health ED	-0.03 [-0.1,0.0]	0.06
**Schizophrenia**			
Number of Visits			
	Hospitalization	-0.01 [-0.0,0.0]	0.70
	Mental Health Hospitalizations	-0.01 [0.0,0.0]	0.47
	ED	-0.23 [-0.4,-0.1]	0.01
	Mental Health ED	-0.04 [-0.1,0.0]	0.02

© 2023 Lewis A et al. *JAMA Health Forum*.

eTable 19. Restricted patient population whose VBP exposure status is consistent throughout study period: Differential change in primary outcomes after VBP for VBP attributed patients

		Estimates (95% CI)	p-value
Depression			
Number of Visits			
	Behavioral Health	1.12 [0.7,1.5]	<0.001
	Primary Care	1.58 [1.0,2.1]	<0.001
Bipolar Depression			
Number of Visits			
	Behavioral Health	1.18 [0.3,2.0]	0.007
	Primary Care	2.62 [1.7,3.5]	<0.001
Schizophrenia			
Number of Visits			
	Behavioral Health	-0.35 [-1.5,0.8]	0.56
	Primary Care	1.79 [0.9,2.7]	<0.001

© 2023 Lewis A et al. *JAMA Health Forum*.

eTable 20. Restricted patient population whose VBP exposure status is consistent throughout study period: Differential change in secondary outcomes after VBP for VBP attributed patients

		Estimates (95% CI)	p-value
Depression			
Number of Visits			
	Hospitalization	-0.01 [0.0,0.0]	0.15
	Mental Health Hospitalizations	-0.01 [0.0,0.0]	<0.001
	ED	-0.07 [-0.1,0.0]	0.06
	Mental Health ED	0 [0.0,0.0]	0.41
Bipolar Depression			
Number of Visits			
	Hospitalization	-0.02 [-0.1,0.0]	0.36
	Mental Health Hospitalizations	-0.01 [0.0,0.0]	0.30
	ED	-0.03 [-0.2,0.1]	0.69
	Mental Health ED	0.01 [0.0,0.0]	0.66
Schizophrenia			
Number of Visits			
	Hospitalization	0 [-0.1,0.1]	0.95
	Mental Health Hospitalizations	-0.01 [0.0,0.0]	0.59
	ED	-0.29 [-0.6,0.0]	0.06
	Mental Health ED	0 [0.0,0.0]	0.90

© 2023 Lewis A et al. *JAMA Health Forum*.

eTable 21. Differential change in quality outcomes after VBP for VBP attributed patients

	Mean visits per year					
	VBP		non-VBP		Adjusted	
	Before VBP	After VBP	Before VBP	After VBP	Estimates (95% CI)	p-value
**Depression**						
Number of Visits						
Preventative Hospitalizations	0.24	0.20	0.20	0.17	0.00 [-0.01,0.01]	0.75
Readmissions	0.10	0.08	0.08	0.07	-0.01 [-0.01,0.0001]	0.05
**Bipolar**						
Number of Visits						
Preventative Hospitalizations	0.38	0.31	0.36	0.30	-0.01 [-0.03,0.02]	0.63
Readmissions	0.19	0.15	0.17	0.14	-0.01 [-0.03,0.01]	0.18
**Schizophrenia**						
Number of Visits						
Preventative Hospitalizations	0.48	0.37	0.48	0.38	-0.02 [-0.05,0.01]	0.13
Readmissions	0.24	0.18	0.24	0.19	-0.01 [-0.03,0.01]	0.32

© 2023 Lewis A et al. *JAMA Health Forum*.

eTable 22. Differential change in Combined Outpatient Utilization after VBP for VBP attributed patients

	Mean visits per year				Regression Results			
	VBP		non-VBP		Unadjusted		Adjusted	
	Before VBP	After VBP	Before VBP	After VBP	Estimates (95% CI)	p-value	Estimates (95% CI)	p-value
**Depression**								
Number of Visits								
Combined	12.1	14.5	10.4	12.2	0.64 [-0.05,1.33]	0.07	0.54 [-0.15,1.24]	0.13
**Bipolar**								
Number of Visits								
Combined	15.8	20.2	13.6	17.5	0.46 [-0.87,1.79]	0.50	0.29 [-1.10,1.68]	0.68
**Schizophrenia**								
Number of Visits								
Combined	17.4	20.1	14.4	18.0	-0.96 [-2.44,0.52]	0.20	-1.06 [-2.62,0.51]	0.19

© 2023 Lewis A et al. *JAMA Health Forum.*

eTable 23. Differential change in Mental Health Primary Care (MHPC) visits after VBP for VBP attributed patients

	Mean visits per year				Regression Results	
	VBP		non-VBP		Adjusted	
	Before VBP	After VBP	Before VBP	After VBP	Estimates (95% CI)	p-value
**Depression**						
Number of Visits						
MHPC	0.09	0.07	0.13	0.08	0.02 [0.01,0.04]	0.001
**Bipolar**						
Number of Visits						
MHPC	0.11	0.07	0.14	0.08	0.02 [0.003,0.04]	0.02
**Schizophrenia**						
Number of Visits						
MHPC	0.09	0.06	0.11	0.07	0.01 [-0.01, 0.03]	0.29

© 2023 Lewis A et al. *JAMA Health Forum*.

eTable 24. Multiple Comparison Adjustment for primary and secondary outcomes

	Estimates (95% CI)	Adjusted p-value	Adjusted significance level
**Depression**			
Behavioral health	0.91 [0.5,1.3]	0.004	0.021
Primary care	-0.36 [-0.9,-0.2]	0.04	0.021
Mental Health ED	-0.008 [-0.015, -0.002]	0.013	0.021
Mental Health Hospitalization	-0.01 [-0.013, -0.003]	0.008	0.021
**Bipolar Depression**			
Behavioral health	1.01 [0.22,1.8]	0.017	0.021
Primary care	-1.51 [-1.7,0.28]	0.03	0.021
Mental Health ED	-0.02 [-0.04, -0.001]	0.03	0.021
Mental Health hospitalization	-0.01 [-0.02, 0.01]	0.04	0.021
**Schizophrenia**			
Behavioral health	0.26 [-0.71.2,]	0.05	0.021
Primary care	-1.31 [-2.5,-0.116]	0.03	0.021
Mental Health ED	-0.04 [-0.06, -0.01]	0.021	0.021
Mental Health hospitalization	-0.01 [-0.03, 0.01]	0.05	0.021

© 2023 Lewis A et al. *JAMA Health Forum*.

eTable 25. NYC vs non-NYC: Differential change in primary outcomes after VBP for VBP patients

		**NYC population**: Change in outcomes trends VBP patients post-VBP reform		**non-NYC population:** Change in outcomes trends for VBP patients post-VBP reform	
		Estimates (95% CI)	p-value	Estimates (95% CI)	p-value
Depression					
Number of Visits					
	Behavioral health	1.3 [0.4,2.2]	0.003	0.64 [0.3,1.0]	<0.001
	Primary care	-0.35 [-1.4,0.7]	0.50	-0.91 [-1.2,-0.6]	<0.001
Bipolar Depression					
Number of Visits					
	Behavioral health	1.23 [-0.2,2.7]	0.09	1.02 [0.3,1.8]	0.01
	Primary care	-1.07 [-3.2,1.0]	0.32	-1.51 [-2.0,-1.0]	<0.001
Schizophrenia					
Number of Visits					
	Behavioral health	0.69 [-0.9,2.3]	0.40	0.01 [-1.1,1.1]	0.99
	Primary care	-1.92 [-4.3,0.5]	0.11	-1.74 [-2.4,-1.1]	<0.001

© 2023 Lewis A et al. *JAMA Health Forum*.

eTable 26. NYC vs non-NYC: Differential change in secondary outcomes after VBP for VBP patients

		**NYC population**: Change in outcomes trends VBP patients post-VBP reform		**non-NYC population:** Change in outcomes trends for VBP patients post-VBP reform	
		Estimates (95% CI)	p-value	Estimates (95% CI)	p-value
**Depression**					
Number of Visits					
	Hospitalization	-0.02 [-0.0,0.0]	0.07	0 [-0.0,0.0]	0.51
	Mental Health Hospitalizations	-0.01 [-0.0,-0.0]	0.001	-0.01 [-0.0,0.0]	0.11
	ED	-0.07 [-0.2,0.0]	0.15	-0.1 [-0.2,-0.0]	0.02
	Mental Health ED	-0.01 [-0.0,0.0]	0.08	0 [-0.0,0.0]	0.35
**Bipolar**					
Number of Visits					
	Hospitalization	-0.01 [-0.1,0.0]	0.61	0 [-0.0,0.0]	0.99
	Mental Health Hospitalizations	-0.01 [-0.0,0.0]	0.32	0 [-0.0,0.0]	0.87
	ED	-0.15 [-0.3,0.0]	0.13	-0.16 [-0.3,0.0]	0.06
	Mental Health ED	-0.03 [-0.1,0.0]	0.16	-0.01 [-0.0,0.0]	0.41
**Schizophrenia**					
Number of Visits					
	Hospitalization	-0.02 [-0.1,0.0]	0.45	0 [-0.0,0.0]	0.88
	Mental Health Hospitalizations	-0.01 [-0.0,0.0]	0.30	-0.01 [-0.0,0.0]	0.71
	ED	-0.14 [-0.4,0.1]	0.28	-0.28 [-0.5,-0.1]	0.01
	Mental Health ED	-0.03 [-0.1,0.0]	0.15	-0.04 [-0.1,0.0]	0.09

© 2023 Lewis A et al. *JAMA Health Forum*.

eTable 27. Differential change in outcomes after VBP for Schizophrenia VBP patients stratified by Charlson Co-Morbidity Score

	Categorical Charlson Co-morbidity Score											
	0-2			3-4			5-7			8+		
	Mean	Estimates (95% CI)	p-value	Mean	Estimates (95% CI)	p-value	Mean	Estimates (95% CI)	p-value	Mean	Estimates (95% CI)	p-value
Primary Outcomes												
Mental Health	8.00	0.07 [-0.87,1.0]	0.89	9.44	1.4 [-0.36,3.16]	0.12	9.76	0.61 [-1.62,2.8]	0.59	11.07	-0.51 [-2.9,1.88]	0.68
Primary Care	7.39	-1.31 [-2.1,-0.49]	0.002	12.74	-1.51 [-3.6,0.60]	0.16	11.87	-1.4 [-3.54,0.75]	0.20	17.20	-1.28 [-4.9,2.3]	0.49
Secondary Outcomes												
Hospitalizations	0.53	0 [-0.03,0.03]	0.90	0.89	-0.05 [-0.14,0.04]	0.29	0.88	0.02 [-0.08,0.11]	0.72	1.52	-0.08 [-0.22,0.06]	0.25
Mental Health Hospitalizations	0.28	-0.01 [-0.03,0.01]	0.38	0.28	-0.02 [-0.08,0.04]	0.51	0.29	0.04 [0.00,0.09]	0.08	0.30	-0.03 [-0.09,0.02]	0.19
Emergency Room Visits	1.52	-0.14 [-0.29,0.01]	0.08	2.42	-0.23 [-0.67,0.21]	0.31	2.10	-0.25 [-0.68,0.18]	0.25	3.18	-0.56 [-1.06-0.06]	0.03
Mental Health Emergency Room Visits	0.27	-0.03 [-0.06,0.01]	0.10	0.28	0 [-0.06,0.06]	0.94	0.27	-0.09 [-0.19,0.00]	0.06	0.28	-0.05 [0.11,0.01]	0.13

© 2023 Lewis A et al. *JAMA Health Forum*.

eReferences

1. Health, N.D.o. *MEDICAID ENCOUNTER DATA REPORTING FOR APD AND MMCOR CATEGORY OF SERVICE* 2022; Available from: https://www.health.ny.gov/health_care/managed_care/reports/.
2. Hea;th, N.Y.D.o. *HEALTH.DATA.NY.GOV*. 2015; Available from: https://health.data.ny.gov/browse?sortBy=alpha.
3. Health, N.Y.D.o. *Safety Net Determinations*. 2019; Available from: https://www.health.ny.gov/health_care/medicaid/redesign/dsrip/safety_net/safety_net_determinations.htm.
4. Hospitals., N.A.o.P., *Ambulatory Care Source Book: Findings from the 2001 NAPH ambulatory care survey.* . Washington, DC: National Association of Public Hospitals and Health Systems, 2001.
5. Au, M., et al., *Attribution in DSRIP Demonstration Programs: A Spotlight on New Jersey and New York*. 2017, Mathematica Policy Research.
6. Cost, H. and U. Project, *Clinical classifications software (CCS) for ICD-10.* Rockville, MD: Agency for Healthcare Research and Quality, 2012.
7. Heslin, K.C., A. Elixhauser, and C.A. Steiner, *Hospitalizations involving mental and substance use disorders among adults, 2012: statistical brief# 191.* 2015.
8. Melfi, C.A. and T.W. Croghan, *Use of claims data for research on treatment and outcomes of depression care.* Med Care, 1999. **37**(4 Suppl Lilly): p. As77-80.
9. Schmidt, E.M., et al., *Potentially preventable medical hospitalizations and emergency department visits by the behavioral health population.* The journal of behavioral health services & research, 2018. **45**(3): p. 370-388.
10. Tamburrino, M.B., et al., *Antidepressant medication adherence: a study of primary care patients.* Primary care companion to the Journal of clinical psychiatry, 2009. **11**(5): p. 205.
11. Medford-Davis, L.N., et al., *The Role of Mental Health Disease in Potentially Preventable Hospitalizations.* Medical care, 2018. **56**(1): p. 31-38.
12. Samartzis, L. and M.A. Talias, *Assessing and improving the quality in mental health services.* International journal of environmental research and public health, 2020. **17**(1): p. 249.
13. Castillo, E.G., et al., *New York State Medicaid Reforms: Opportunities and Challenges to Improve the Health of Those with Serious Mental Illness.* J Health Care Poor Underserved, 2017. **28**(3): p. 839-852.
14. Weller, W., E. Martin, and D. Boyd, *Final Summative Report by the Independent Evaluator for the New York State Delivery System Reform Incentive Payment (DSRIP) Program*. 2019, Retrieved from Albany, NY: https://www. health. ny. gov/health_care/medicaid ….
15. Doktorchik, C., et al., *Validation of a case definition for depression in administrative data against primary chart data as a reference standard.* BMC Psychiatry, 2019. **19**(1): p. 9.
16. Rokicki, S., et al., *Trends in postpartum mental health care before and during COVID-19.* Health Services Research, 2022. **57**(6): p. 1342-1347.
17. Benjamini, Y. and D. Yekutieli, *The control of the false discovery rate in multiple testing under dependency.* Annals of statistics, 2001: p. 1165-1188.

© 2023 Lewis A et al. *JAMA Health Forum*.

18. Frimpong, E.Y., et al., *Impact of the 1115 behavioral health Medicaid waiver on adult Medicaid beneficiaries in New York State.* Health Services Research, 2021. **56**(4): p. 677-690.
19. Ko, H. and S.A. Glied, *Associations Between a New York City Paid Sick Leave Mandate and Health Care Utilization Among Medicaid Beneficiaries in New York City and New York State.* JAMA Health Forum, 2021. **2**(5): p. e210342-e210342.
20. Grove, L.R., et al., *Evaluating the potential for primary care to serve as a mental health home for people with schizophrenia.* Gen Hosp Psychiatry, 2017. **47**: p. 14-19.

© 2023 Lewis A et al. *JAMA Health Forum*.
