# Supplementary material for: Medicaid Value-Based Payments and Health Care Use for Patients With Mental Illness
Source: JAMA Health Forum. 2023 Sep 22;4(9):e233197. doi: 10.1001/jamahealthforum.2023.3197 (PMC10517380; doi:10.1001/jamahealthforum.2023.3197)
Supplement: Supplement 2. — Data Sharing Statement [file jamahealthforum-e233197-s002.pdf]

Data Sharing Statement

Lewis. Medicaid Value-Based Payments and Health Care Use for Patients With Mental Illness. *JAMA Health Forum*. Published September 22, 2023. doi:10.1001/jamahealthforum.2023.3197

Data
Data available: No

Additional Information
Explanation for why data not available: Our primary data source is Medicaid administrative claims, which has protected patient data and is not distributable
